# Physical recovery across care pathways up to 12 months after hospitalization for COVID-19: A multicenter prospective cohort study (CO-FLOW)

**DOI:** 10.1016/j.lanepe.2022.100485

**Published:** 2022-08-24

**Authors:** Julia C. Berentschot, Majanka H. Heijenbrok-Kal, L. Martine Bek, Susanne M. Huijts, Jasper van Bommel, Michel E. van Genderen, Joachim G.J.V. Aerts, Gerard M. Ribbers, Merel E. Hellemons, Rita J.G. van den Berg-Emons, CO-FLOW Collaboration Group, Joachim G.J.V. Aerts, L. Martine Bek, Julia C. Berentschot, Rita J.G. van den Berg-Emons, Sieshem Bindraban, Wouter J.B. Blox, Jasper van Bommel, Shai A. Gajadin, Michel E. van Genderen, Diederik A.M.P.J. Gommers, Majanka H. Heijenbrok-Kal, Merel E. Hellemons, Roxane Heller, Susanne M. Huijts, Erwin Ista, Stephanie van Loon-Kooij, Rutger Osterthun, Laurien Oswald, Gerard M. Ribbers, Ronald N. van Rossem, Herbert J. van de Sande, Rob Slingerland, Robert van der Stoep, Janette J. Tazmi-Staal, Marieke M. Visser, Markus P.J.M. Wijffels, Eva G. Willems

**Affiliations:** a Department of Respiratory Medicine, Erasmus MC, University Medical Center Rotterdam, the Netherlands; b Department of Rehabilitation Medicine, Erasmus MC, University Medical Center Rotterdam, the Netherlands; c Rijndam Rehabilitation, Rotterdam, the Netherlands; d Department of Adult Intensive Care Medicine, Erasmus MC, University Medical Center Rotterdam, the Netherland

**Keywords:** COVID-19, Physical function, Physical recovery, Rehabilitation

## Abstract

**Backgroud:**

The sudden COVID-19 pandemic forced quick development of care pathways for patients with different needs. Trajectories of physical recovery in hospitalized patients for COVID-19 following different care pathways are unknown. We aimed to assess trajectories of physical recovery and levels of physical function reached within the different care pathways. Additionally, we assessed differences in physical function across care pathways at follow-up visits.

**Methods:**

This multicenter prospective cohort study of adults who had been hospitalized for COVID-19 was performed in 10 centers, including 7 hospitals (1 academic and 6 regional hospitals) and 3 rehabilitation centers (1 medical rehabilitation center and 2 skilled nursing facilities), located in the Netherlands. Study visits were performed at 3, 6, and 12 months post-hospital discharge and included assessment of cardiorespiratory fitness (6 min walk test [6MWT], 1 min sit-to-stand test [1MSTST]), muscle strength (maximum handgrip strength [HGS]) and mobility (de Morton Mobility Index [DEMMI]).

**Findings:**

We report findings for 582 patients who had been discharged from hospital between March 24, 2020 and June 17, 2021. Patients had a median age of 60·0 years, 68·9% (401/582) were male, 94·6% (561/582) had received oxygen therapy, and 35·2% (205/582) mechanical ventilation. We followed patients across four different rehabilitation settings: no rehabilitation (No-rehab, 19·6% [114/582]), community-based rehabilitation (Com-rehab, 54·1% [315/582]), medical rehabilitation (Med-rehab, 13·7% [80/582]), and rehabilitation in a skilled nursing facility (SNF-rehab, 12·5% [73/582]). Overall, outcomes in 6MWT (14·9 meters [95% CI 7·4 to 22·4]), 1MSTST (2·2 repetitions [1·5 to 2·8]), and HGS (3·5 kg [2·9 to 4·0]) improved significantly (*p<*0·001) from 3 to 6 months and only HGS from 6 to 12 months (2·5 kg [1·8 to 3·1]; *p<*0·001). DEMMI scores did not significantly improve over time. At 3 months, percentage of normative values reached in 1MSTST differed significantly (*p<*0.001) across care pathways, with largest impairments in Med- and SNF-rehab groups. At 12 months these differences were no longer significant, reaching, overall, 90·5% on 6MWD, 75·4% on 1MSTST, and 106·9% on HGS.

**Interpretation:**

Overall, physical function improved after hospitalization for COVID-19, with largest improvement within 6 months post-discharge. Patients with rehabilitation after hospital discharge improved in more than one component of physical function, whereas patients without rehabilitation improved solely in muscle strength. Patients who received rehabilitation, and particularly patients with Med- and SNF-rehab, had more severe impairment in physical function at 3 months, but reached equal levels at 12 months compared to patients without follow-up treatment. Our findings indicate the importance of rehabilitation.

**Funding:**

ZonMw, Rijndam Rehabilitation, Laurens (The Netherlands).

**Research in context**

**Evidence before this study**

We searched PubMed for follow-up studies on long-term physical recovery using objective measurements in patients who had been hospitalized for COVID-19, published between Jan 1, 2021 and April 13, 2022, without language restrictions. The following search terms were used: (“COVID-19” OR “SARS-CoV-2” OR “Coronavirus disease 2019”) AND “hospital*” AND (“long-term*” OR “recovery*” OR “persistent” OR “follow*” OR “sequelae”) AND (“physic*” OR “fitness” OR “rehabilitation”) AND (“cohort” or “observational”). One large Asian cohort study (>1000 patients) and other smaller studies reported physical outcomes up to 1 year after hospitalization for COVID-19. In the large cohort study from Wuhan, China, cardiorespiratory fitness was measured with the 6 min walk test at 6 and 12 months follow-up, reporting overall good physical recovery at 12 months. Physical recovery across patients who received different rehabilitative care after hospital discharge has not yet been reported.

**Added value of this study**

To our knowledge, this is the first study that evaluates physical recovery after hospitalization for COVID-19 across patients who followed different care pathways. We followed patients with no follow-up treatment, community-based rehabilitation, medical rehabilitation, and rehabilitation in a skilled nursing facility after hospital discharge. In our Dutch multicenter prospective cohort study, objective assessment of physical function was performed at 3, 6, and 12 months after hospital discharge.

This study shows that physical function, comprising cardiorespiratory fitness, muscle strength, and mobility, improved after hospitalization for COVID-19, with largest improvement achieved within 6 months after hospital discharge. Patients who received rehabilitation, and particularly patients with Med- and SNF-rehab, had more severe impairment in physical function at 3 months but reached equal levels at 12 months compared to patients without follow-up treatment.

**Implications of all the available evidence**

Our findings contain valuable information for both health care professionals and patients in the convalescent phase after acute SARS-CoV-2 infection. Patients who required more intensive rehabilitative care returned to physical levels that were comparable to less affected patients without follow-up treatment. Our findings indicate the importance of rehabilitation.

Alt-text: Unlabelled box

## Introduction

The clinical spectrum of coronavirus disease-2019 (COVID-19) ranges from asymptomatic infection to critical illness requiring admission to an intensive care unit (ICU). Although COVID-19 primarily affects the respiratory system, many organs can be affected and a wide range of post-acute sequelae may occur, including impaired physical function.^1^, ^2^, ^3^, ^4^, ^5^ Post COVID-19 condition, as defined by the World Health Organization, occurs in individuals with a history of probable or confirmed SARS-CoV-2 infection, usually 3 months from the onset of COVID-19 with symptoms that last for at least 2 months and are not explained by an alternative diagnosis.^6^ These symptoms may be new onset or persist from acute illness and may fluctuate or relapse over time. Based on self-reported measures, 49% to 92% of patients hospitalized for COVID-19 experienced one or more persistent symptoms at 12 months follow-up.^2^^,^^7^, ^8^, ^9^ Regarding physical symptoms, we previously reported that 63% of COVID-19 patients experienced deconditioning (exertional dyspnea), 41% muscle weakness, and 43% balance problems 12 months after hospital discharge.^9^

Objective and longitudinal data on long-term physical recovery after hospitalization for COVID-19 are scarce. Prior studies mostly focused on cardiorespiratory fitness, reporting 80-110% of predicted levels at 12 months after hospitalization.^2^^,^^10^, ^11^, ^12^ However, other components of physical function such as muscle strength and mobility are also important constructs in the evaluation of physical recovery. For example, among non-COVID-19 patients, after admission for acute respiratory distress syndrome, patients still experienced muscle wasting and weakness 12 months after discharge from ICU.^13^ Thus, an objective assessment of different components of physical function is needed to obtain in-depth information on long-term physical recovery after COVID-19.

The sudden pandemic forced quick development of care pathways for COVID-19 patients, pathways initially based on inadequate knowledge of patient aftercare needs. Fortunately, as we now know, most hospitalized patients are sufficiently independent at hospital discharge and able to return home with or without support of community-based rehabilitation.^14^ However, some patients are referred to medical rehabilitation, often severely affected younger patients with a high premorbid functional level, or to a skilled nursing facility in case of vulnerable patients with a low premorbid functional level.^15^^,^^16^ Trajectories of physical recovery in patients related to different care pathways have not been assessed to date. This knowledge is important to gain insight into whether care pathways have to be optimized to provide the right physical care for different needs.

The primary study aim was to assess trajectories of physical recovery and levels of physical function reached within the different care pathways. We objectively assessed physical function, comprising cardiorespiratory fitness, muscle strength, and mobility, at 3, 6, and 12 months after hospital discharge. The secondary study aim was to assess differences in physical function across care pathways at follow-up visits. We hypothesized that patients who require more intensive rehabilitation have more impaired physical function at 3 months after hospital discharge and that these differences in physical function reduce over time.

## Methods

### Study design and population

This study is part of an ongoing two-year prospective multicenter cohort study, “COVID-19 Follow-up care paths and Long-term Outcomes Within the Dutch health care system” (CO-FLOW), in the Rotterdam–Rijnmond–Delft region of The Netherlands.^17^ The study was performed in 10 centers, including 7 hospitals (1 academic and 6 regional hospitals) and 3 rehabilitation centers (1 medical rehabilitation center and 2 skilled nursing facilities), all located in this region. Patients with COVID-19 who were discharged from one of the participating hospitals were invited to participate in study visits at the outpatient clinic of hospitals if they met the following criteria: 1) COVID-19 diagnosis based on positive reverse transcription polymerase chain reaction, or based on multidisciplinary team decision concerning symptoms and chest computed tomography scan or positive serology; 2) aged 18 years or older; 3) within 6 months, but preferably within 3 months, after hospital discharge; 4) patient or relative has sufficient knowledge of the Dutch or English language.^17^ Incapacitated patients were not included, patients were considered non-capable if they were cognitively impaired (e.g. dementia) and therefore unable to understand instructions to perform study measurements. Patients received study information from their pulmonary physician during regular follow-up, or by invitation letter, and for patients with inpatient rehabilitation this was done by the rehabilitation physician or elderly care physician. Recruitment of study participants occurred independent of the patient's recovery status; this was largely based on availability of research personnel to recruit patients and to perform study visits. If patients consented they were contacted by the researchers to schedule the study visit. All 650 participants in the CO-FLOW study provided written informed consent before the start of the measurements.^17^ This study was approved by the Medical Ethics Committee of Erasmus MC (MEC-2020-0487) and registered in The Netherlands Trial Register (no. NL8710). More detailed information about the CO-FLOW study protocol is published elsewhere.^17^ Here we present a pre-planned interim analysis on physical outcomes of patients who attended at least one follow-up visit at 3, 6, or 12 months after hospital discharge.

### Study procedure

Study visits were scheduled around 3, 6, and 12 months after hospital discharge and when possible alongside the clinical follow-up for COVID-19 in the participating hospitals (supplementary figure S1). Patients who were discharged from clinical follow-up were invited to visit Erasmus MC for the remaining study visits; we arranged a home visit for patients who were unwilling or unable to visit Erasmus MC.^17^ During study visits, patients performed non-invasive clinical tests of physical function. Leisure time physical activity level (inactive, light, regular, or hard) before COVID-19 infection was measured with the Saltin–Grimby Physical Activity Level Scale questionnaire.^18^ Demographic characteristics, such as migration background, education level, and employment status, and rehabilitative care after hospital discharge were collected with a face-face interview, electronic patient records, and the iMTA Medical Cost Questionnaire.^19^ Regarding rehabilitation, during the face-to-face interview patients were asked whether they had received rehabilitative care for COVID-19 illness and, if so, the type of treatment, care setting, and the duration of inpatient rehabilitation. All study visits were conducted by a small team of junior researchers, assistants, and medical students, who all received training from experienced senior researchers. Clinical characteristics during hospital admission, such as treatment, ICU admission, and length of hospital stay, were retrospectively collected from electronic patient records in the participating hospitals. All collected data were stored in the Castor Electronic Data Capture system (Castor EDC, Amsterdam, the Netherlands).

### Care pathways

**Figure 1:**
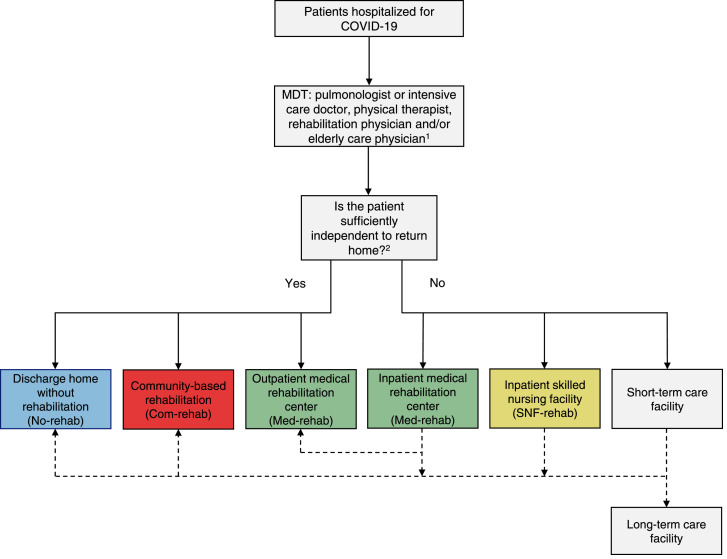
**Dutch care pathways for hospitalized COVID-19 patients**. MDT: multidisciplinary team. ^1^Assessment of functional impairments (physical, cognitive, and/or psychological), medical status, care needs, comorbidity, and premorbid functional level.^15^^2^Rehabilitation as defined by the World Health Organization aims to help a child, adult, or older person to be as independent as possible in everyday activities and enables participation in education, work, recreation, and meaningful life roles such as taking care of family.^20^ Geriatric rehabilitation focuses primarily on frail elderly with co-morbidities. Medical rehabilitation is aimed at high-intensity treatment, mostly of a younger population.

Patients who are sufficiently independent at hospital discharge are discharged home without rehabilitation or with support of community-based rehabilitation or outpatient medical rehabilitation. Patients unable to be discharged home and who require more intensive rehabilitative care are referred to inpatient medical rehabilitation center or to a skilled nursing facility.^20^ We followed patients in four care pathways comprising different post-acute care settings: 1) no rehabilitation (No-rehab), 2) community-based rehabilitation (Com-rehab), 3) in- and/or outpatient medical rehabilitation (Med-rehab), and 4) inpatient rehabilitation in a skilled nursing facility (SNF-rehab). We followed patients in the four different care pathways and categorized them based on the most specialized aftercare they had received after hospitalization for COVID-19, with Med- and SNF-rehab being the most specialized. None of the participants received both Med- and SNF-rehab. The Dutch care pathways, including rehabilitation triage, for hospitalized COVID-19 patients are presented in Figure 1 and programs across the different rehabilitation services are reported below.^15^^,^^16^

*No-rehab*: Patients are sufficiently recovered and do not require rehabilitation.
*Com-rehab*: Outpatient treatment to support recovery to premorbid functional levels. This often comprises monodisciplinary treatment once or twice a week, such as physical therapy or occupational therapy, of varying duration ranging from weeks to months.
*Med-rehab*: Intensive in- or outpatient multidisciplinary treatment to reduce functional deficits and to support recovery to premorbid function levels, aiming to return home in the case of inpatient rehabilitation. The type and duration of treatment is based on patient-centered functional goal setting and varies across patients. The program is guided by an interdisciplinary team of a rehabilitation physician, nurse, physiotherapist, occupational therapist, psychologist, speech- and language therapist, movement therapist, social worker, and dietician, depending on the patients care needs. Treatment during inpatient rehabilitation is often provided 4-5 times per day for approximately 4-6 weeks. The duration of outpatient treatment is usually 8-12 weeks. After inpatient rehabilitation, patient may continue outpatient med-rehab or community-based rehabilitation.
*SNF-rehab*: Moderately intensive inpatient multidisciplinary treatment to reduce functional deficits and dependency and to support recovery to premorbid function levels, aiming to return home. The type and duration of treatment is based on patient-centered functional goal setting and varies across patients. The program is guided by an interdisciplinary team of an elderly care physician, nurse, physiotherapist, occupational therapist, psychologist, speech- and language therapist, movement therapist, social worker, and dietician, depending in the patients care needs. During inpatient rehabilitation, treatment is provided for a maximum of 5 times a week for 4-8 weeks. After inpatient rehabilitation, patient may continue outpatient SNF-rehab or community-based rehabilitation.

### Physical outcome measures

*Cardiorespiratory fitness* was measured with the submaximal 6 min walk test (6MWT) and 1 min sit-to-stand test (1MSTST), which both involve functional performance. For practical reasons the 6MWT was not assessed in patients who were visited at home. During these tests the participants were allowed to rest or stop if needed. During the 6MWT, participants were instructed to walk as far and as fast as possible back and forth along a 30- or 20 m corridor, depending on the test location, with verbal encouragement provided after approximately every minute.^21^ Oxygen saturation during the 6MWT was recorded using a fingertip pulse oximeter. Exercise-induced desaturation was indicated by a decrease of ≥4% upon 6MWT.^22^ Outcome of the 6MWT was the distance walked in meters (6MWD), which was also normalized to percentage of normative values and to performance below the lower limit of normal (LLN) according to sex-, age-, height-, and weight-stratified equations described by Enright and Sherrill.^23^

As a secondary measure of cardiorespiratory fitness, all participants performed the 1MSTST.^24^ Participants started in a seated position (standard chair, 46 cm) and were instructed to perform as many repetitions of sit-to-stand as possible in one minute without using arm support. Outcome of the 1MSTST was the number of sit-to-stand (STS) repetitions, and these counts were also normalized to percentage of normative values according to sex- and age-stratified reference values as described by Strassmann and colleagues.^25^ We included the 1MSTST to facilitate measurement of cardiorespiratory fitness in participants who are unable to perform the 6MWT and for the participants we studied at home. Outcomes on the 1MSTST are strongly correlated with those on the 6MWT in patients with limitations due to pulmonary disease.^26^^,^^27^

*Muscle strength* was assessed by measurement of maximum isometric handgrip strength (HGS) in kg, using the Jamar hydraulic handheld dynamometer. HGS is considered as an indicator of overall muscle strength.^28^ Participants were tested in a sitting position with their feet flat on the floor, shoulder in an adducted position and elbow at 90 degrees. They performed three attempts per hand with approximately 30 sec of rest in between. Arm support was provided to those who were unable to hold the dynamometer without support. We used the maximum HGS measured over six attempts (3 per arm) as outcome measure, and HGS was also expressed as percentage of normative values according to sex- and age-stratified reference values, and as performance below the cutoff for weak HGS, defined as <27 kg in men and <16 kg in women.^29^^,^^30^

*Mobility* was measured with the de Morton Mobility Index (DEMMI) test, originally developed to measure mobility in elderly hospitalized patients and also validated in an ICU population.^31^^,^^32^ The DEMMI test consists of 15 items administered from easiest as follows: tasks in bed (3 items), tasks in a chair (3 items), static balance (4 items), walking (2 items), and dynamic balance (2 items). The raw sum score ranges from 0 to 19 and is then converted into an interval score ranging from 0 to 100, where higher scores represent better mobility.

### Statistical analysis

Continuous variables are presented as median and interquartile range (IQR), Shapiro–Wilk tests indicated that all continuous variables were not normally distributed, and categorical variables as a number and percentage. To assess differences in demographic and clinical characteristics during hospital admission across care pathways (No-, Com-, Med-, and SNF-rehab) we performed a χ2 test or Kruskal–Wallis test, as appropriate, and a Bonferroni correction was applied for multiple testing (significance level set at *p<*0.001). For the primary aim, we used generalized estimating equations (GEE) with repeated measurements to explore the trajectories of physical outcomes (6MWT, 1MSTST, HGS, and DEMMI) over time in the total cohort and within care pathways in separated analyses. The GEE approach considers within-subject correlations and uses all available measurements despite incomplete data. All GEE analyses were performed using an unstructured correlation matrix. For the assessment of physical recovery in the total cohort we entered measurement time (3, 6, and 12 months) as fixed factor in each GEE analysis. For the assessment of physical recovery within care pathways we entered care pathway as fixed factor, the interaction of time and care pathway, and adjusted for demographic (age, sex, and employment status) and clinical (having one or more comorbidities, obesity, delirium, thrombotic event, admission to intensive care unit, and the length of hospital stay) characteristics during hospital admission in each GEE analysis. Missing data in categorical covariates were analyzed in the category no or unknown (no or unknown versus yes). For the covariate obesity we first imputed missing body mass index (BMI) values with the median BMI value within care pathways, as appropriate, and values were then dichotomized (obese if BMI≥30 kg/m²). Likewise, we used GEE analysis to assess the trajectories of the percentages of normative values reached in 6MWT, 1MSTST, and HGS over time; no appropriate normative values for DEMMI are available for our sample. These GEE models were adjusted for the same covariates as previously mentioned, excluding age and sex and in case of the 6MWT also obesity (normative values are already adjusted for these characteristics). We used least significant difference post hoc tests for pairwise comparisons between follow-up visits in the total cohort and within care pathways. We also performed similar GEE analyses to asses trajectories of physical outcomes and percentage of normative values reached in physical tests within care pathways without adjustment for covariates, see Supplementary Figures S2 and S3. For the secondary aim, we performed a cross-sectional data analysis to assess whether the percentage of normative values reached in 6MWT, 1MSTST, and HGS differed across care pathways at each time point. These outcomes were obtained from post hoc tests using the previously described GEE analyses adjusted for covariates. The GEE results are presented as the estimated mean and standard error (SE) as well as estimated mean difference between time points and their 95% confidence interval (95% CI). The source data of physical outcomes are presented in Supplementary Table S2 for the total cohort and in Supplementary Table S3 stratified according to care pathway. The level of statistical significance was set *p<*0·05 unless stated otherwise. All statistical analyses were performed with IBM SPSS Statistics version 28 (SPSS Inc., Chicago, IL, USA).

### Role of the funding source

Funders of the study had no role in study design, data collection, data analysis, interpretation, or writing of the report.

## Results

### Study population

**Figure 2:**
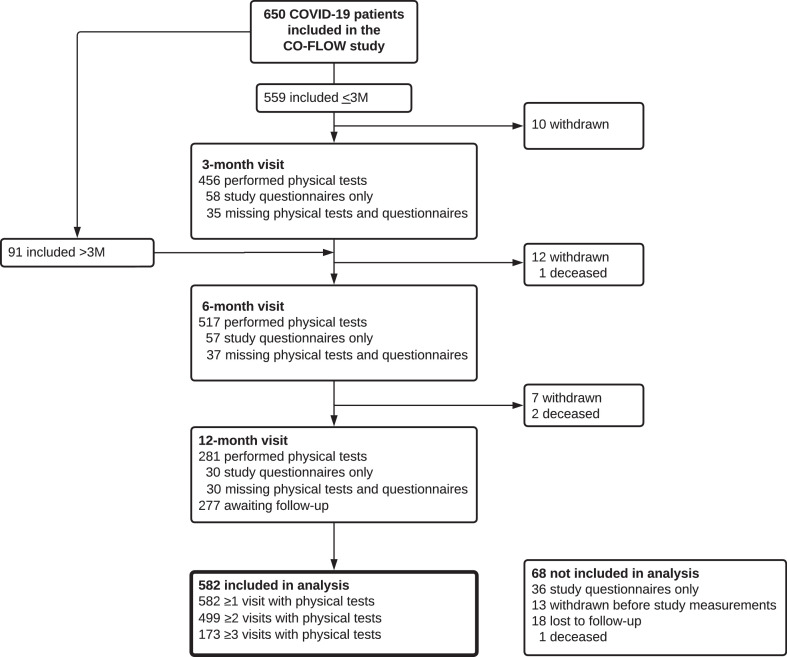
**Flowchart of CO-FLOW study participants included in the analysis.** In total 582 patients attended at least one follow-up visit with physical tests and were included in this analysis. ≤3M refers to participants enrolled prior to or at 3 months after hospital discharge; >3M refers to participants enrolled after 3 months but within 6 months after hospital discharge.

Between July 1, 2020, and Sept 9, 2021, 650 patients who were hospitalized for COVID-19 were prospectively enrolled in the CO-FLOW study (Figure 2). All patients were discharged from hospital between March 24, 2020 and June 17, 2021. The total number of patients hospitalized for COVID-19 during the recruitment period in the region was 4569 of whom 1199 (26%) died during hospitalization.^33^ The number of patients that had been invited is largely unknown due to logistical reasons. From the 3370 survivors, 650 patients (19% of all survivors) were included in this study. As of this interim analysis at Dec 3, 2021, 582 patients attended ≥1 study visit and were included in this analysis. The proportion of patients with ≥1 comorbidities was slightly lower and the length of hospital stay shorter in patients who were included in this analysis compared to those who were not (Supplementary Table S1).

In total, 114 (19·6%) patients did not participate in follow-up treatment (No-rehab), 315 (54·1%) received Com-rehab, 80 (13·7%) received Med-rehab, and 73 (12·5%) received SNF-rehab. The majority of Com-rehab patients (291/315, 92·4%) received physiotherapy. In Med-rehab, 67 patients (83·8%) received inpatient rehabilitation for a median stay of 32.0 (IQR 25·0-42·0) days, of whom 10 (14·9%) patients continued outpatient Med-rehab after discharge; 13 (16·2%) patients received only outpatient treatment. All 73 (100%) patients in SNF-rehab received inpatient rehabilitation, for a median stay of 29·5 (IQR 18·5-39·0) days; 3 (4·2%) patients continued outpatient SNF-rehab after discharge.

Table 1 presents the demographic and clinical characteristics during hospital admission in the total cohort and stratified according to care pathway. Among all 582 patients, the median age was 60·0 (53·0-67·0), 31·1% were female, and the median length of hospital stay was 12·0 (IQR 6·0-27·0) days. Significant differences existed among patients across the different care pathways. Compared to the other care pathways, the SNF-rehab group was significantly older (67·0 [60·5-73·0] years), the No-rehab group had fewer comorbidities (67·3% had ≥1 comorbidities), and the Med-rehab group was characterized by a high proportion of patients with obesity (58·8%). Patients in both the Med- and SNF-rehab groups were characterized by worse clinical characteristics; the majority of these patients were admitted to an ICU and they required a significantly longer hospital stay than patients in the No- and Com-rehab groups.

**Table 1:** Demographic and clinical characteristics during hospital admission for COVID-19 in the total cohort and stratified according to care pathway.

	n^a^	All (*n=*582)	No-rehab (*n=* 114)	Com-rehab (*n=* 315)	Med-rehab (*n=* 80)	SNF-rehab (*n=* 73)	*p* value^e^
**Demographic characteristics**							
Median (IQR) age, years	-	60·0 (53·0–67·0)	59·0 (51·0–68·0)	60·0 (53·0–67·0)	57·0 (53·0–62·8)	67·0 (60·5–73·0)	<0·001*
Sex	-						0·018
Female		181 (31·1)	30 (26·3)	115 (36·5)	17 (21·3)	19 (26·0)	
Male		401 (68·9)	84 (73·7)	200 (63·5)	63 (78·8)	54 (74·0)	
Median (IQR) BMI, kg/m²	69	29·3 (25·7–32·1)	27·9 (24·7–31·0)	28·1 (25·8–32·0)	30·0 (27·5–33·8)	27·7 (25·6–33·2)	<0·001*
*Comorbidities*	-						
≥1		473 (81·3)	76 (67·3)	266 (84·4)	66 (82·5)	65 (89·0)	<0·001*
Obesity (BMI≥30 kg/m²)		224 (38·5)	31 (27·2)	119 (37·8)	47 (58·8)	27 (37·0)	<0·001*
Diabetes		117 (20·1)	20 (17·5)	65 (20·6)	12 (15·0)	20 (27·4)	0·24
Cardiovascular disease and/or hypertension		225 (38·7)	32 (28·1)	125 (39·7)	29 (36·3)	39 (53·4)	0·006
Pulmonary disease		145 (24·9)	17 (14·9)	88 (27·9)	20 (25·0)	20 (27·4)	0·05
Renal disease		52 (8·9)	9 (7·9)	32 (10·2)	4 (5·0)	7 (9·6)	0·51
Gastrointestinal disease		30 (5·2)	6 (5·3)	16 (5·1)	7 (8·8)	1 (1·4)	0·24
Neurological disease		60 (10·3)	8 (7·0)	30 (9·5)	8 (10·0)	14 (19·2)	0·05
Malignancy		65 (11·2)	9 (7·9)	38 (12·1)	8 (10·0)	10 (13·7)	0·56
Autoimmune disease		61 (10·5)	11 (9·6)	32 (10·2)	7 (8·8)	11 (15·1)	0·57
Mental disorder		29 (5·0)	3 (2·6)	16 (5·1)	6 (7·5)	4 (5·5)	0·49
*Migration background*	3						0·49^b^
European		415 (71·7)	76 (67·9)	234 (74·3)	54 (68·4)	51 (69·9)	
Dutch Caribbean		80 (13·8)	18 (16·1)	38 (12·1)	12 (15·2)	12 (16·4)	
Asian		36 (6·2)	6 (5·4)	15 (4·8)	8 (10·1)	7 (9·6)	
Turkish		25 (4·3)	7 (6·3)	13 (4·1)	3 (3·8)	2 (2·7)	
(North) African		23 (4·0)	5 (4·5)	15 (4·8)	2 (2·5)	1 (1·4)	
*Education level*	7						0·34
Low		201 (35·0)	40 (35·7)	107 (34·1)	23 (29·9)	31 (43·1)	
Middle		202 (35·1)	38 (33·9)	108 (34·4)	31(40·3)	25 (34·7)	
High		172 (29·9)	34 (30·4)	99 (31·5)	23 (29·9)	16 (22·2)	
*Smoking status*	4						0·12^b^
Never		254 (43·9)	53 (47·3)	140 (44·4)	38 (48·7)	23 (31·5)	
Former		313 (54·2)	56 (50·0)	168 (53·3)	39 (50·0)	50 (68·5)	
Current		11 (1·9)	3 (2·7)	7 (2·2)	1 (1·3)	0 (0·0)	
*Physical activity level*^c^	5						0·20
Inactive		76 (13·2)	15 (13·4)	40 (12·7)	7 (9·1)	14 (19·2)	
Light		305 (52·9)	56 (50·0)	164 (52·1)	44 (57·1)	41 (56·2)	
Regular		159 (27·6)	30 (26·8)	93 (29·5)	21 (27·3)	15 (20·5)	
Hard		37 (6·4)	11 (9·8)	18 (5·7)	5 (6·5)	3 (4·1)	
Employed	6	344 (59·7)	67 (59·8)	181 (57·8)	68 (88·3)	28 (37·8)	<0·001*
**Clinical characteristics**							
PCR-confirmed COVID-19 infection	-	572 (98·3)	108 (94·7)	313 (99·4)	79 (98·8)	72 (98·6)	
Other confirmed COVID-19 infection^d^	-	10 (1·7)	6 (5·3)	2 (0·6)	1 (1·3)	1 (1·4)	
Thrombotic event	13	89 (15·6)	8 (7·1)	34 (11·0)	27 (34·6)	20 (28·6)	<0·001*
Delirium	17	144 (25·5)	17 (14·9)	39 (12·7)	50 (66·7)	38 (55·1)	<0·001*
Oxygen supplementation	-	561 (96·4)	106 (93·0)	304 (96·5)	80 (100·0)	71 (97·3)	na
HFNC	37	177 (32·5)	19 (17·9)	81 (27·5)	37 (52·1)	40 (54·8)	<0·001*
ICU admission	-	237 (40·7)	19 (16·7)	85 (27·0)	76 (95·0)	57 (78·1)	<0·001*
IMV	-	205 (35·2)	12 (10·5)	65 (20·6)	73 (91·3)	55 (75·3)	<0·001*
Median (IQR) duration of IMV, days	8	14·0 (8·0–26·5)	8·5 (6·0–17·8)	8·0 (6·0–13·8)	23·5 (14·3–33·8)	15·0 (10·0–32·0)	<0·001*
Tracheostomy	11	73 (12·8)	3 (2·7)	12 (3·8)	36 (46·8)	22 (31·9)	<0·001*
Median (IQR) LOS ICU, days	4	16 (9·0–30·0)	8·0 (4·0–14·0)	9·0 (6·3–15·0)	28·0 (18·0–39·0)	20·0 (13·0–39·0)	<0·001*
*COVID-19 directed treatment*	31						0·23^b^
None		132 (24·0)	32 (29·1)	62 (20·3)	19 (27·9)	19 (28·4)	
Steroids		388 (70·4)	71 (64·5)	228 (74·5)	41 (60·3)	48 (71·6)	
Antivirals		80 (14·5)	24 (21·8)	50 (16·3)	5 (7·4)	1 (1·5)	
Anti-inflammatory		66 (12·0)	3 (2·7)	33 (10·9)	13 (19·1)	17 (25·4)	
Hydroxy)chloroquine		16 (2·9)	5 (4·5)	5 (1·6)	6 (8·8)	0 (0·0)	
Convalescent plasma		8 (1·5)	2 (1·8)	4 (1·3)	0 (0·0)	2 (3·0)	
Median (IQR) LOS hospital, days	1	12 (6·0–27·0)	7·0 (4·0–10·5)	9·0 (5·0–16·0)	43·0 (30·5–54·8)	29·0 (21·5–45·0)	<0·001*
**Time interval from discharge to follow-up visit, days**							
Median (IQR) 3 months	-	93·0 (88·0–101·0)	92·0 (88·0–101·3)	94·0 (88·0–102·0)	93·0 (88·0–100·0)	92·0 (88·0–101·3)	0·99
Median (IQR) 6 months	-	184·0 (180·0–192·0)	183·0 (180·0–191·0)	184·0 (180·0–192·0)	186·0 (178.8–195·3)	182·0 (179·0–193·0)	0·88
Median (IQR) 12 months	-	366·0 (362·0–372·0)	365·0 (360·0–372·0)	366·0 (361·3–372·0)	365·5 (361·8–371.3)	365·5 (363·0–381·3)	0·32

Data are presented as n (%) unless stated otherwise. Care pathways comprise patients with No-rehab: no rehabilitation, Com-rehab: community-based rehabilitation, Med-rehab: in- and outpatient medical rehabilitation, and SNF-rehab: inpatient rehabilitation in a skilled nursing facility after hospitalization for COVID-19. IQR: interquartile range; BMI: body mass index; PCR: polymerase chain reaction; HFNC=high flow nasal cannula; ICU=intensive care unit; IMV=invasive mechanical ventilation; LOS=length of stay; na=not applicable.

a In case of missing data the number of missing data are presented.

b Due to small group sizes we assessed differences in migration background as European vs non-European, in smoking status as never vs ever, and in COVID-19 directed treatment as none vs any treatment.

c Leisure time physical activity level was measured with the Saltin–Grimby Physical Activity Level Scale questionnaire.^27^

d COVID-19 diagnosis based on multidisciplinary team decision concerning symptoms and chest computed tomography scan or positive serology.

e Obtained using a χ2 test or Kruskal–Wallis test, as appropriate. Statistically significant p value after Bonferroni correction (*p<*0.001) is denoted by *.

### Overall trajectories of physical function

Table 2 shows the GEE outcomes of physical tests at follow-up visits in the total cohort.

**Table 2:** Physical function in COVID-19 patients at 3, 6, and 12 months after hospital discharge.

	3 months	6 months	12 months	Mean difference 3–6 months (95% CI), *p* value	Mean difference 6–12 months (95% CI), *p* value	Mean difference 3–12 months (95% CI), *p* value
**Cardiorespiratory fitness**						
*6MWT*						
6MWD, m	476·0 (5·3)	490·9 (5·3)	495·2 (5·6)	14·9 (7·4–22·4), <0·001	4·3 (−3·4–12·1), 0·3	19·2 (10·4–28·0), <0·001
6MWD, %pred^a^	87·8 (1·0)	89·8 (0·9)	90·5 (1·0)	2·0 (0·4–3·6), 0·01	0·7 (−0·8–2·3), 0·4	2·8 (0·9–4·6), 0·004
*1MSTST*						
STS repetitions, n	24·9 (0·5)	27·1 (0·5)	27·7 (0·6)	2·2 (1·5–2·8), <0·001	0·6 (−0·2–1·5), 0·2	2·8 (1·8–3·8), <0·001
STS repetitions, %pred^b^	67·1 (1·2)	72·9 (1·2)	75·4 (1·5)	5·8 (4·0–7·6), <0·001	2·5 (0·07–5·0), 0·04	8·3 (5·6–121·1), <0·001
**Muscle strength**						
*HGS*						
Maximum, kg	35·5 (0·6)	39·0 (0·6)	41·4 (0·6)	3·5 (2·9–4·0), <0·001	2·5 (1·8–3·1), <0·001	5·9 (5·1–6·7), <0·001
Maximum, %pred^c^	91·1 (1·0)	100·2 (1·0)	106·9 (1·2)	9·1 (7·7–10·5), <0·001	6·6 (4·9–8·4), <0·001	15·7 (13·7–17·7), <0·001
**Mobility**						
*DEMMI*						
Total score	88·0 (0·6)	88·7 (0·6)	89·4 (0·7)	0·7 (−0·4–1·7), 0·2	0·7 (−0·5–1·9), 0·3	1·4 (0·05–2·7), 0·04

Data are presented as estimated mean (standard error) unless stated otherwise, obtained from generalized estimating equations analysis. The number of patients included in the analysis for 6MWT: 537, 1MSTST: 567, HGS: 577, and DEMMI: 573. 95% CI=95% confidence interval; 6MWT=6 min walk test; 6MWD=6 min walk distance; %pred=percentage of normative value; 1MSTST=1 min sit-to-stand test; STS: sit-to-stand; HGS=handgrip strength; DEMMI=de Morton Mobility Index.

a Calculated using reference equations described by Enright and Sherill.^18^

b Reference values described by Strassman and colleagues.^20^

c Reference values described by Dodds and colleagues.^23^

*Cardiorespiratory fitness:* A total of 58, 87, and 54 patients did not perform the 6MWT at 3, 6, and 12 months, respectively, due to logistical reasons such as home visits or patients were physically unable to perform the 6MWT. At 3 months the estimated mean 6MWD was 476·0 m (SE 5·3) and 87·8% (1·0) of norm; the number of STS repetitions was 24·9 (0·5) and 67·1% (1·2) of norm. Both 6MWD and STS repetitions improved significantly from 3 to 6 months, but not thereafter (Table 2). The proportion of patients with a 6MWD result below the LLN was 21·4% (81/379) at 3 months, 16·5% (73/442) at 6 months, and 16·8% (40/238) at 12 months (supplementary table S2). At 12 months, patients performed 90·5% (1·0) of normative 6MWD and 75·4% (1·5) of normative STS repetitions.

*Muscle strength:* At 3 months the estimated mean HGS was 35·5 (SE 0·6) kg and 91·1% (1·0) of norm. HGS improved significantly from 3 to 6 months as well as from 6 to 12 months (Table 2). The proportion of patients with weak HGS decreased from 11·5% (51/442) at 3 months to 6·3% (32/512) at 6 months and to 5·7% (16/280) at 12 months (supplementary table S2). Patients performed 106·9% (1·2) of normative HGS at 12 months.

*Mobility:* At 3 months the estimated mean DEMMI score was 88·0 (SE 0·6) points. DEMMI scores did not significantly improve over time (Table 2).

### Trajectories of physical function within care pathways

**Figure 3:**
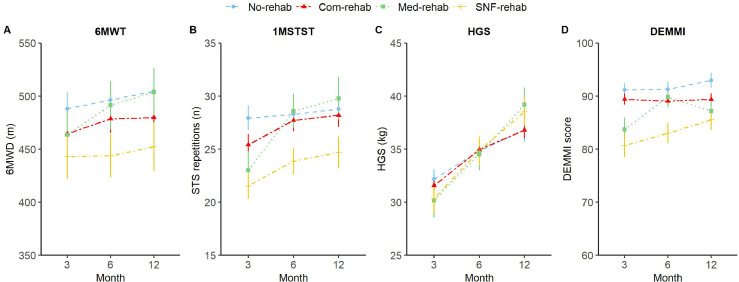
**Trajectories of outcomes in 6MWT, 1MSTST, HGS, and DEMMI over time within care pathways assessed at 3, 6, and 12 months after hospital discharge**. Care pathways comprise patients with No-rehab: no rehabilitation, Com-rehab: community-based rehabilitation, Med-rehab: in- and outpatient medical rehabilitation, and SNF-rehab: inpatient rehabilitation in a skilled nursing facility after hospitalization for COVID-19. Trajectories of physical outcomes over time were assessed with generalized estimating equations analysis, adjusted for demographic and clinical characteristics during hospital admission including age, sex, having one or more comorbidities, obesity, employment status, delirium, thrombotic event, admission to intensive care unit, and the length of hospital stay. Data are presented as estimated mean with standard error. In 6MWT: significant improvement in Com-rehab (*p*=0·01) and Med-rehab (*p*=0·047) from 3 to 6 months but not thereafter; no significant improvement over time within other care pathways. In 1MSTST: significant improvement in Com-rehab (*p<*0·001), Med-rehab (*p<*0·001), and SNF-rehab (*p*=0·002) from 3 to 6 months but not thereafter; no significant improvement over time within No-rehab. In HGS: significant improvement within all care pathways from 3 to 6 months and from 6 to 12 months (all *p<*0·001 except for No-rehab from 6 to 12 months [*p*=0·002]). In DEMMI: significant improvement in Med-rehab (*p*=0·001) from 3 to 6 months but not thereafter; no significant improvement over time within other care pathways. 6MWT=6 min walk test; 6MWD=6 min walk distance; 1MSTST=1 min sit-to-stand test; STS=sit-to-stand; HGS=handgrip strength; DEMMI=de Morton Mobility Index.

**Figure 4:**
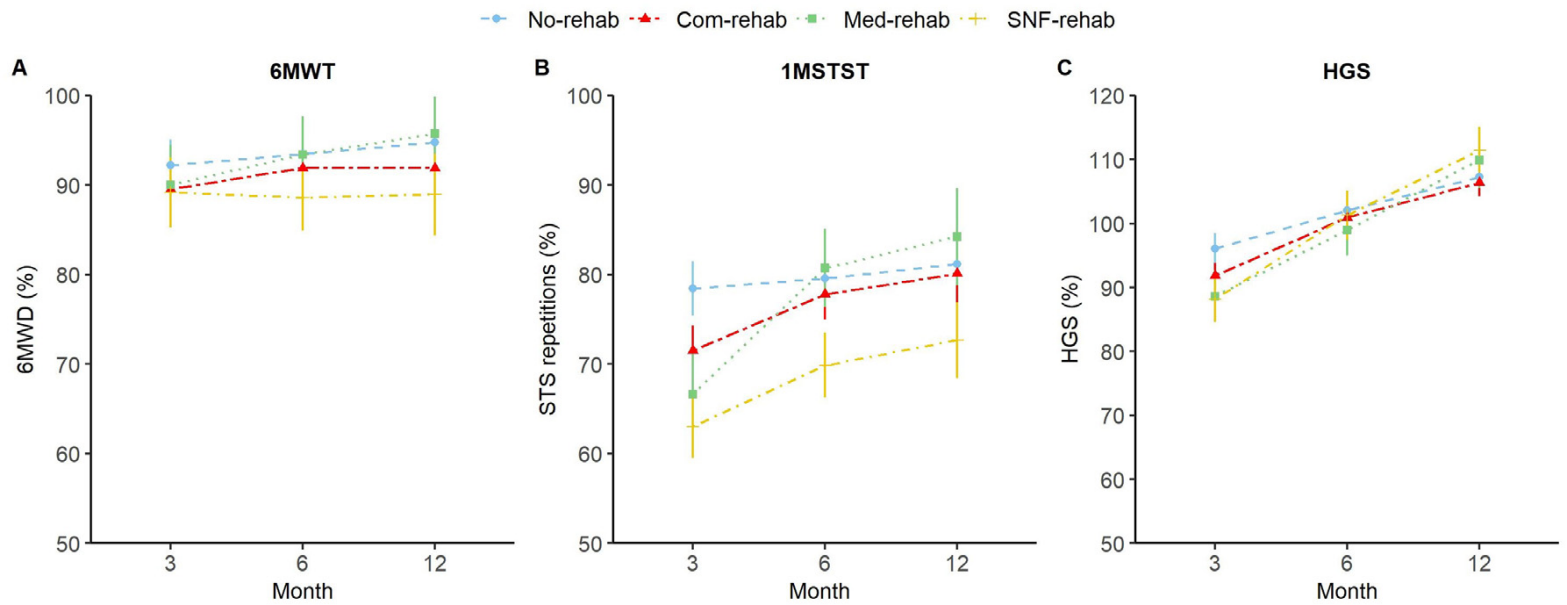
**Trajectories of the percentage of normative values reached in 6MWT, 1MSTST, and HGS over time within care pathways assessed at 3, 6, and 12 months after hospital discharge**. Care pathways comprise patients with No-rehab: no rehabilitation, Com-rehab: community-based rehabilitation, Med-rehab: in- and outpatient medical rehabilitation, and SNF-rehab: inpatient rehabilitation in a skilled nursing facility after hospitalization for COVID-19. The percentages of normative values reached in physical tests were assessed with generalized estimating equations analysis, adjusted for demographic and clinical characteristics during hospital admission including having one or more comorbidities, obesity (excluded in 6MWT analysis), employment status, delirium, thrombotic event, admission to intensive care unit, and the length of hospital stay. Data are presented as estimated mean with standard error. In 6MWT: significant improvement in Com-rehab (*p=*0·03) from 3 to 6 months but not thereafter; no significant improvement within other care pathways. In 1MSTST: significant improvement in Com-rehab (*p<*0·001), Med-rehab (*p<*0·001), and SNF-rehab (*p=*0·001) from 3 to 6 months but not thereafter; no significant improvement over time within No-rehab. In HGS: significant improvement within all care pathways from 3 to 6 months and from 6 to 12 months (all *p<*0·001 except for No-rehab 6-12 months [*p=*0·002]). Normative values in 6MWT are calculated using reference equations described by Enright and Sherill,^23^ in 1MSTST using reference values described by Strassman and colleagues,^25^ and in HGS using reference values described by Dodds and colleagues.^29^ 6MWT=6 min walk test; 6MWD=6 min walk distance; 1MSTST=1 min sit-to-stand test; STS=sit-to-stand; HGS=handgrip strength.

The trajectories of physical function and percentage of normative values reached within care pathways are graphically presented in Figure 3, Figure 4, the outcomes of GEE analyses are presented per physical test in Supplementary Tables S4-S7.

*Cardiorespiratory fitness:* The 6MWD improved significantly in Com- and Med-rehab from 3 to 6 months but not thereafter; no improvement was found within No- and SNF-rehab (Figure 3A). Likewise, the number of STS repetitions improved in both Com- and Med-rehab as well as in SNF-rehab from 3 to 6 months, but not thereafter (Figure 3B). Similar trends were found in the trajectories of the percentage of normative values over time within care pathways (Figures 4A and 4B).

*Muscle strength:* HGS improved significantly in all care pathways from 3 to 6 months as well as from 6 to 12 months (Figure 3). More than 100% of normative HGS was reached within all care pathways at 12 months follow-up (Figure 4C). Similar trends were found in the trajectories of the percentage of normative HGS over time within care pathways (Figure 4C).

*Mobility:* DEMMI scores improved significantly in Med-rehab from 3 to 6 months but not thereafter; no significant improvement was found within other care pathways (Figure 3D).

### Comparison of physical function across care pathways

*Cardiorespiratory fitness:* The percentage of norm reached in the number of STS repetitions differed significantly between care pathways at follow-up, but not in 6MWD. At 3 months, Com-rehab (estimated mean difference −6·9% [95% CI −12·9 to −1·0]; *p*=0·02)), Med-rehab (−11·8% [−21·9 to −1·8]; *p*=0·02), and SNF-rehab (−15·4% [−23·7 to −7·2]; *p<*0·001)) had a significantly lower percentage of STS repetitions than No-rehab, as well as for SNF-rehab compared to Com-rehab (−8·5% [−15·6 to −1·4]; *p*=0·02); outcomes did not differ significantly between other care pathways. At 6 months SNF-rehab had a significantly lower percentage of normative STS repetitions than No-rehab (−9·7% [−18·0 to −1·3]; *p*=0·02), Com-rehab (−7·9% [−15·3 to −0·6]; *p*=0·04), and Med-rehab (−10·9% [−19·3 to −2·4]; *p*=0·01); outcomes did not differ significantly between other care pathways. At 12 months these differences across care pathways were no longer significant in STS repetitions.

*Muscle strength:* The percentage of normative HGS did not significantly differ between care pathways at follow-up visits. However, outcomes of the GEE analysis without adjustment for covariates showed lower percentages of normative HGS in Med- and SNF-rehab than in No- and Com-rehab (supplementary figure S3).

## Discussion

This study provides objective measurements of long-term physical recovery after hospitalization for COVID-19 among patients who followed different care pathways. The study showed that cardiorespiratory fitness improved from 3 to 6 months solely in patients with rehabilitative care after hospital discharge, mobility improved only in Med-rehab from 3 to 6 months, whereas muscle strength improved within all care pathways from 3 to 6 months as well as from 6 to 12 months. The study also showed that the patients who received rehabilitation, and particularly patients with Med- or SNF-rehab, started off worse but reached at 12 months levels of physical function equal to those of less affected patients, indicating the importance of rehabilitation. At 12 months, overall, patients reached 91% of normative 6MWD, 75% of normative STS repetitions, and 107% of normative HGS.

Earlier studies on long-term physical recovery after hospitalization for COVID-19 are limited. Wu et al. reported improvement in 6MWD not only from 3 to 6 months, as in our study, but also from 6 to 12 months after hospital discharge.^12^ However, in contrast to our cohort, patients with comorbidities and invasive mechanical ventilation were not included, whereas the overall length of hospital stay for COVID-19 was longer. Also, it is unclear if patients received rehabilitative care after hospital discharge and, if so, in what context. Other studies found no improvement in 6MWD over 12 months follow-up, which could be due to small sample sizes,^10^^,^^11^ or between 6 and 12 months.^2^ Recovery in HGS was assessed only in a cohort of patients admitted to ICU for COVID-19, indicating improved HGS over 12 months follow-up.^11^ At 12 months, their patients achieved lower HGS (37 kg) compared to our patients (41 kg), but the study included only critically ill patients whereas our cohort comprised patients from both wards and ICUs.

Physical recovery occurred particularly in patients with rehabilitative care after hospital discharge, showing clinically meaningful outcomes in cardiorespiratory fitness. Bohannon and Crouch suggested that changes between 14·0 and 30·5 m in 6MWD can be considered clinically important (minimal clinically important difference, MCID).^34^ Both Med- and SNF-rehab groups exceeded 14·0 m in 6MWD from 3 to 6 months. In 1MSTST, only patients with Med-rehab exceeded the MCID of 3 repetitions.^27^ Although we found that the improvement in HGS was statistically significant within all care pathways, these changes may not be considered as clinically meaningful (MCID 5·0-6·5 kg).^35^ Noteworthy, the literature does not identify a clear MCID for HGS and more studies are needed. Furthermore, it should be realized that the first study measurements were performed 3 months after hospital discharge and physical recovery within the first 3 months after hospital discharge was not assessed. Our findings imply both statistical significance and clinical meaningful outcomes in cardiorespiratory fitness, in particular in Med-rehab, and underline the importance of rehabilitation.

Our results seem to show that rehabilitation triage was successful, with more intensive rehabilitation provided to most impaired patients after hospitalization for COVID-19. Triage is the process of evaluating patients in relation to clinical, social, and affection pre-requisites to enhance the effectiveness of participation in a therapeutic program.^36^ Resources for rehabilitation are limited and the triage process enables the best use of these resources. The fact that the most severely impaired patients were referred to Med-rehab and that these patients showed both statistically significant and clinically meaningful improvements, seems to underscore an effective triage and rehabilitation process. However, our observational cohort design does not allow definite inferences.

At 12 months, overall, patients showed good recovery in cardiorespiratory fitness and muscle strength. These results are noteworthy given the high proportions of patients with comorbidities and severe illness. Still, 17% of patients had a 6MWD result below the LLN at 12 months. It is important to note that the normative values we used for 6MWD are from a healthy population without comorbidities. Therefore, it is not realistic to expect that all patients would reach 100% of norm. However, overall, our patients reached more than 100% of normative HGS and only 6% had impaired HGS at 12 months. The large proportion of patients that received rehabilitative care (80%) may have played a role in this recovery.

Among all patients, only 75% of norm was reached in 1MSTST at 12 months. This is relatively low compared to the achievements on 6MWT and HGS. Although we used the 1MSTST as a secondary outcome measure for cardiorespiratory fitness, the number of 1MSTST repetitions is also related to functional lower muscle strength.^37^ The difference in normative values reached in 6MWT and 1MSTST at 12 months may indicate that there is still some impairment in functional lower muscle strength rather than in cardiorespiratory fitness. This hypothesis is supported by a recent study by Lorent and colleagues, reporting a lower proportion of patients with impaired 6MWD (16/222, 8%) than patients with impaired quadriceps strength (31/222, 32%) at 12 months after hospitalization for COVID-19.^38^ However, because the reference values that we have used for the different physical outcomes were obtained from different study samples, as well as that different reference values for 6MWT were used by Lorent and colleagues,^38^ the findings should be interpreted with caution.

In the last decade there has been growing attention for the functional long-term impairments among survivors of ICU admission, captured under the term post-intensive care syndrome (PICS).^39^ Our patients that had been admitted to the ICU more frequently required more intensive rehabilitation (Med- and SNF-rehab). Not surprisingly, these patients had more impaired physical function at 3 months, but at 12 months these differences caught up. However, at 12 months, impaired 6MWD and HGS remained present in patients across all care pathways. We believe that this underlines that PICS is not unique to intensive care survivors, but long-term functional impairments are part of a continuum of critical illness. Functional impairment in patients that were not admitted to the ICU should be taken as seriously, and all patients should qualify for appropriate rehabilitative care.

This cohort study has several strengths, including the longitudinal and multicenter study design and the objective measurement of varied components of physical function. Also, we included patients who were admitted to either ward or ICU for COVID-19 in The Netherlands. A limitation is that we could not compare our outcomes with pre-morbid levels; therefore, we used normative values of the general population. Unfortunately, normative values on physical tests during the COVID-19 pandemic, including the possible influence of lockdown and restrictive measures, are not available. Furthermore, normative values for the different outcomes are obtained from different reference groups and the normative values for the 6MWT are from 1998, urgently needing revision. However, these normative values have also been used in other COVID-19 studies.^2^^,^^3^^,^^10^ The 650 participants in the CO-FLOW study were recruited from all patients who survived hospitalization and who visited the outpatient clinic for regular COVID-19 follow-up by pulmonary physicians in the participating hospitals. These numbers depended on the local logistics in each hospital, transfers to other regions, and temporary COVID-19 lock-down regulations, in which clinical follow-up was postponed or only performed by phone. Therefore, these numbers are largely unknown, which is a limitation of this study. However, recruitment of study participants occurred independently of the patients recovery status and was largely based on availability of research personnel. Our study contains an overrepresentation of patients (41%) who had been admitted to ICU compared to all hospitalized patients (16%) for COVID-19 in the Netherlands.^40^ Our academic center served as a regional referral center for ICU patients, and many study participants were included from this center. Regarding care pathways, these pathways represent the national strategy of aftercare that was established in the Netherlands and represent hospitalized patients with different disease severity who require different rehabilitation facilities.

In conclusion, this study provides an objective evaluation on physical recovery after hospitalization for COVID-19, following patients across different care pathways. Overall, physical function improved after hospitalization for COVID-19, with largest improvement within 6 months post-discharge. Patients with rehabilitation after hospital discharge improved in more than one component of physical function, whereas patients without rehabilitation improved solely in muscle strength. Patients who received rehabilitation, and particularly patients with Med- or SNF-rehab, had more severe impairment in physical function at 3 months after hospital discharge but reached equal levels at 12 months compared to less affected patients. Future research should look further into refining triage to allocate rehabilitation resources in the best way, finding the most effective rehabilitation programs, and establishing determinants of poor physical recovery.

## Contributors

MH and RBE shared senior authorship and contributed equally to this paper. All authors were involved in the study design and had full access to the data in the study. All authors and members of the CO-FLOW Collaboration Group contributed to the acquisition, analysis, or interpretation of data. JB, MHK, LB, MH, RBE drafted the manuscript. JB, MHK performed the statistical analysis. All authors and members of the CO-FLOW Collaboration Group critically revised and approved the manuscript. MHK, MH, RBE provided supervision.

## Data sharing statement

The datasets that support the findings of this ongoing study are not yet publicly available, but are available from the corresponding author upon reasonable request.

## Declaration of interests

All authors have no conflicts of interest related to this work.

## CO-FLOW Collaboration Group

Joachim G.J.V. Aerts^1^, L. Martine Bek^2^, Julia C. Berentschot^1^, Rita J.G. van den Berg-Emons^2^, Sieshem Bindraban^3^, Wouter J.B. Blox^4^, Jasper van Bommel^5^, Shai A. Gajadin^6^, Michel E. van Genderen^5^, Diederik A.M.P.J. Gommers^5^, Majanka H. Heijenbrok-Kal^2,7^, Merel E. Hellemons^1^, Roxane Heller^8^, Susanne M. Huijts^1^, Erwin Ista^9,10^, Stephanie van Loon-Kooij^11^, Rutger Osterthun^2,7^, Laurien Oswald^3^, Gerard M. Ribbers^2,7^, Ronald N. van Rossem^11^, Herbert J. van de Sande^12^, Rob Slingerland^13^, Robert van der Stoep^14^, Janette J. Tazmi-Staal^15^, Marieke M. Visser^7^, Markus P.J.M. Wijffels^7^, Eva G. Willems^15^

*Affiliations:*

^1^Department of Respiratory Medicine, Erasmus MC, University Medical Center Rotterdam, The Netherlands. ^2^Department of Rehabilitation Medicine, Erasmus MC, University Medical Center Rotterdam, The Netherlands. ^3^Department of Respiratory Medicine, Franciscus Gasthuis & Vlietland, Rotterdam, The Netherlands. ^4^Department of Respiratory Medicine, Albert Schweitzer Hospital, Dordrecht, The Netherlands. ^5^Department of Adult Intensive Care Medicine, Erasmus MC, University Medical Center Rotterdam, The Netherlands. ^6^Department of Respiratory Medicine, IJsselland Hospital, Capelle aan de IJssel, The Netherlands. ^7^Rijndam Rehabilitation, Rotterdam, The Netherlands. ^8^Department of Respiratory Medicine, Ikazia Hospital, Rotterdam, The Netherlands. ^9^Departments of Pediatrics and Pediatric Surgery, Intensive Care Unit, Erasmus MC Sophia Children's Hospital Rotterdam, The Netherlands. ^10^Department of Internal Medicine, section Nursing Science, Erasmus MC, Erasmus University Medical Center Rotterdam, The Netherlands. ^11^Department of Respiratory Medicine, Reinier de Graaf Gasthuis, Delft, The Netherlands. ^12^Aafje Nursing Home, Rotterdam, The Netherlands. ^13^Department of Respiratory Medicine, Maasstad Hospital, Rotterdam, The Netherlands. ^14^Department of Physical Therapy, Erasmus MC, University Medical Center Rotterdam, The Netherlands. ^15^Laurens Intermezzo, Rotterdam, The Netherlands.

## Appendix B Supplementary materials

Image, application 1

## References

[1] Zaim S, Chong JH, Sankaranarayanan V, Harky A. (2020). COVID-19 and multiorgan response. Curr Probl Cardiol.

[2] Huang L, Yao Q, Gu X (2021). 1-year outcomes in hospital survivors with COVID-19: a longitudinal cohort study. Lancet North Am Ed.

[3] Belli S, Balbi B, Prince I (2020). Low physical functioning and impaired performance of activities of daily life in COVID-19 patients who survived hospitalisation. Eur Respir J.

[4] Nalbandian A, Sehgal K, Gupta A (2021). Post-acute COVID-19 syndrome. Nat Med.

[5] Paneroni M, Simonelli C, Saleri M (2021). Muscle strength and physical performance in patients without previous disabilities recovering from COVID-19 pneumonia. Am J Phys Med Rehabil.

[6] Soriano JB, Murthy S, Marshall JC, Relan P, Diaz JV (2022). Condition WHOCCDWGoP-C-. A clinical case definition of post-COVID-19 condition by a Delphi consensus. Lancet Infect Dis.

[7] Seeßle J, Waterboer T, Hippchen T (2021). Persistent symptoms in adult patients 1 year after coronavirus disease 2019 (COVID-19): a prospective cohort study. Clin Infect Dis.

[8] Wynberg E, van Willigen HDG, Dijkstra M (2021). Evolution of COVID-19 symptoms during the first 12 months after illness onset. Clin Infect Dis.

[9] LM Bek, JC Berentschot, MH Heijenbrok-Kal, et al. Symptoms persisting after hospitalization for COVID-19: 12 months interim results of the COFLOW study. *medRxiv*. doi: 10.1101/2021.12.11.21267652 (preprint: 13 Dec 2021). PMC9421428 36284829

[10] Betschart M, Rezek S, Unger I (2021). One year follow-up of physical performance and quality of life in patients surviving COVID-19: a prospective cohort study. Swiss Med Wkly.

[11] Latronico N, Peli E, Calza S (2021). Physical, cognitive and mental health outcomes in 1-year survivors of COVID-19-associated ARDS. Thorax.

[12] Wu X, Liu X, Zhou Y (2021). 3-month, 6-month, 9-month, and 12-month respiratory outcomes in patients following COVID-19-related hospitalisation: a prospective study. Lancet Respiratory Med.

[13] Herridge MS, Cheung AM, Tansey CM (2003). One-year outcomes in survivors of the acute respiratory distress syndrome. N Engl J Med.

[14] Wahlgren C, Divanoglou A, Larsson M (2022). Rehabilitation needs following COVID-19: Five-month post-discharge clinical follow-up of individuals with concerning self-reported symptoms. EClinicalMedicine.

[15] Richtlijnendatabase. Indicatiestelling revalidatietraject en hertriage. 2021.https://richtlijnendatabase.nl/richtlijn/covid-19/startpagina_-_langdurige_klachten_en_revalidatie_na_covid-19/startpagina_-_revalidatie_na_covid-19/indicatiestelling_revalidatietraject_en_hertriage_bij_covid-19.html. Accessed 16 June 2022.

[16] Verenso. Behandeladvies Geriatrische Revalidatiezorg (GR). 01-12-2020. https://www.verenso.nl/themas-en-projecten/infectieziekten/covid-19-coronavirus/behandeladvies-gr. Accessed 16 June 2022.

[17] Bek LM, Berentschot JC, Hellemons ME (2021). CO-FLOW: Covid-19 follow-up care paths and long-term outcomes within the Dutch health care system: study protocol of a multicenter prospective cohort study following patients 2 years after hospital discharge. BMC Health Serv Res.

[18] Grimby G, Börjesson M, Jonsdottir IH, Schnohr P, Thelle DS, Saltin B. (2015). The “Saltin–Grimby physical activity level scale” and its application to health research. Scand J Med Sci Sports.

[19] Bouwmans C, Krol M, Severens H, Koopmanschap M, Brouwer W, Hakkaart-van Roijen L. (2015). The iMTA productivity cost questionnaire: a standardized instrument for measuring and valuing health-related productivity losses. Value Health.

[20] WHO. Rehabilitation. 2021. https://www.who.int/news-room/fact-sheets/detail/rehabilitation. Accessed 16 June 2022.

[21] Laboratories ATSCoPSfCPF (2002). ATS statement: guidelines for the six min walk test. Am J Respir Crit Care Med.

[22] Poulain M, Durand F, Palomba B (2003). 6 min walk testing is more sensitive than maximal incremental cycle testing for detecting oxygen desaturation in patients with COPD. Chest.

[23] Enright PL, Sherrill DL. (1998). Reference equations for the six min walk in healthy adults. Am J Respir Crit Care Med.

[24] Koufaki P, Mercer TH, Naish PF. (2002). Effects of exercise training on aerobic and functional capacity of end-stage renal disease patients. Clin Physiol Funct Imaging.

[25] Strassmann A, Steurer-Stey C, Dalla Lana K (2013). Population-based reference values for the 1-min sit-to-stand test. Int J Public Health.

[26] Ozalevli S, Ozden A, Itil O, Akkoclu A. (2007). Comparison of the sit-to-stand test with 6 min walk test in patients with chronic obstructive pulmonary disease. Respir Med.

[27] Crook S, Büsching G, Schultz K (2017). A multicentre validation of the 1-min sit-to-stand test in patients with COPD. Eur Respir J.

[28] Bohannon RW (2015). Muscle strength: clinical and prognostic value of hand-grip dynamometry. Curr Opin Clin Nutr Metab Care.

[29] Dodds RM, Syddall HE, Cooper R (2014). Grip strength across the life course: normative data from twelve British studies. PLoS One.

[30] Cruz-Jentoft AJ, Bahat G, Bauer J (2019). Sarcopenia: revised European consensus on definition and diagnosis. Age Ageing.

[31] de Morton NA, Davidson M, Keating JL. (2008). The de morton mobility index (DEMMI): an essential health index for an ageing world. Health Quality Life Outcomes.

[32] Sommers J, Vredeveld T, Lindeboom R, Nollet F, Engelbert RHH, van der Schaaf M. (2016). de morton mobility index is feasible, reliable, and valid in patients with critical illness. Phys Ther.

[33] Rijksinstituut voor Volksgezondheid en Milieu. Covid-19 cumulatieve aantallen per gemeente. 2022. https://data.overheid.nl/dataset/11508-covid-19-aantallen-gemeente-cumulatief. Accessed 16 June 2022.

[34] Bohannon RW, Crouch R. (2017). Minimal clinically important difference for change in 6 min walk test distance of adults with pathology: a systematic review. J Eval Clin Pract.

[35] Bohannon RW (2019). Minimal clinically important difference for grip strength: a systematic review. J Phys Ther Sci.

[36] Riberto M, Jucá SSH, Miyazaki MH, Battistella LR. (2010). The triage process in rehabilitation centers. Acta Fisiatr.

[37] Bohannon RW, Crouch R. (2019). 1 min sit-to-stand test: systematic review of procedures, performance, and clinimetric properties. J Cardiopul Rehab Prevent.

[38] Lorent N, Vande Weygaerde Y, Claeys E (2022). Prospective longitudinal evaluation of hospitalised COVID-19 survivors 3 and 12 months after discharge. ERJ Open Res.

[39] Paul N, Albrecht V, Denke C, Spies CD, Krampe H, Weiss B. (2022). A decade of post-intensive care syndrome: a bibliometric network analysis. Medicina (Kaunas).

[40] NICE (2022). https://www.stichting-nice.nl/covid-19-op-de-ic.jsp.

