# Supplementary material for: Physical recovery across care pathways up to 12 months after hospitalization for COVID-19: A multicenter prospective cohort study (CO-FLOW)
Source: Lancet Reg Health Eur. 2022 Aug 24;22:100485. doi: 10.1016/j.lanepe.2022.100485 (PMC9402257; doi:10.1016/j.lanepe.2022.100485)
Supplement: Supplementary file 1 [file mmc1.docx]

**Supplementary materials**

Physical recovery across care pathways up to 12 months after hospitalization for COVID-19: a multicenter prospective cohort study (CO-FLOW)

**Authors**

Julia C. Berentschot,^1^ Majanka H. Heijenbrok-Kal,^2,3^ L. Martine Bek,^2^ Susanne M. Huijts,^1^ Jasper van Bommel,^4^ Michel E. van Genderen,^4^ Joachim G.J.V. Aerts,^1^ Gerard M. Ribbers,^2,3^ Merel E. Hellemons,^1^* Rita J.G. van den Berg-Emons,^2^* on behalf of the CO-FLOW Collaboration Group

*Contributed equally

**Affiliations**

^a^ Department of Respiratory Medicine, Erasmus MC, University Medical Center Rotterdam, The Netherlands.

^b^ Department of Rehabilitation Medicine, Erasmus MC, University Medical Center Rotterdam, The Netherlands.

^c^ Rijndam Rehabilitation, Rotterdam, The Netherlands.

^d^ Department of Adult Intensive Care Medicine, Erasmus MC, University Medical Center Rotterdam, The Netherlands.

**Correspondence**: Julia C. Berentschot, Department of Respiratory Medicine, Erasmus MC, Dr. Molewaterplein 40, 3015 GD Rotterdam, The Netherlands, j.berentschot@erasmusmc.nl

| **Table of Contents** | Page |
| --- | --- |
| Supplementary Figure S1. Study timeline | 1 |
| Supplementary Figure S2. Trajectories of outcomes in 6MWT, 1MSTST, HGS, and DEMMI within care pathways at 3, 6, and 12 months after hospital discharge. | 2 |
| Supplementary Figure S3. Trajectories of normative values in 6MWT, 1MSTST and HGS within care pathways assessed at 3, 6, and 12 months after hospital discharge, outcomes of generalized estimating equations analysis. | 3 |
| Supplementary Table S1. Demographic and clinical characteristics of study participants included versus study participants not included in analysis | 4 |
| Supplementary Table S2. Physical function at 3, 6, 12 months after hospital discharge, source data. Values are median (interquartile range) unless stated otherwise | 5 |
| Supplementary Table S3. Physical function across care pathways at 3, 6, and 12 months after hospital discharge, source data. Values are median (interquartile range) unless stated otherwise | 6-7 |
| Supplementary Table S4. 6MWT outcomes across care pathways at 3, 6, and 12 months after hospital discharge. Results from generalized estimating equations analysis, values are estimated mean (standard error) unless stated otherwise | 8 |
| Supplementary Table S5. 1MSTST outcomes across care pathways at 3, 6, and 12 months after hospital discharge. Results from generalized estimating equations analysis, values are estimated mean (standard error) unless stated otherwise | 9 |
| Supplementary Table S6. HGS outcomes across care pathways at 3, 6, and 12 months after hospital discharge. Results from generalized estimating equations analysis, values are estimated mean (standard error) unless stated otherwise | 10 |
| Supplementary Table S7. DEMMI score across care pathways at 3, 6, and 12 months after hospital discharge. Results from generalized estimating equations analysis, values are estimated mean (standard error) unless stated otherwise | 11 |
| References | 12 |

**Supplementary Figure S1**

**
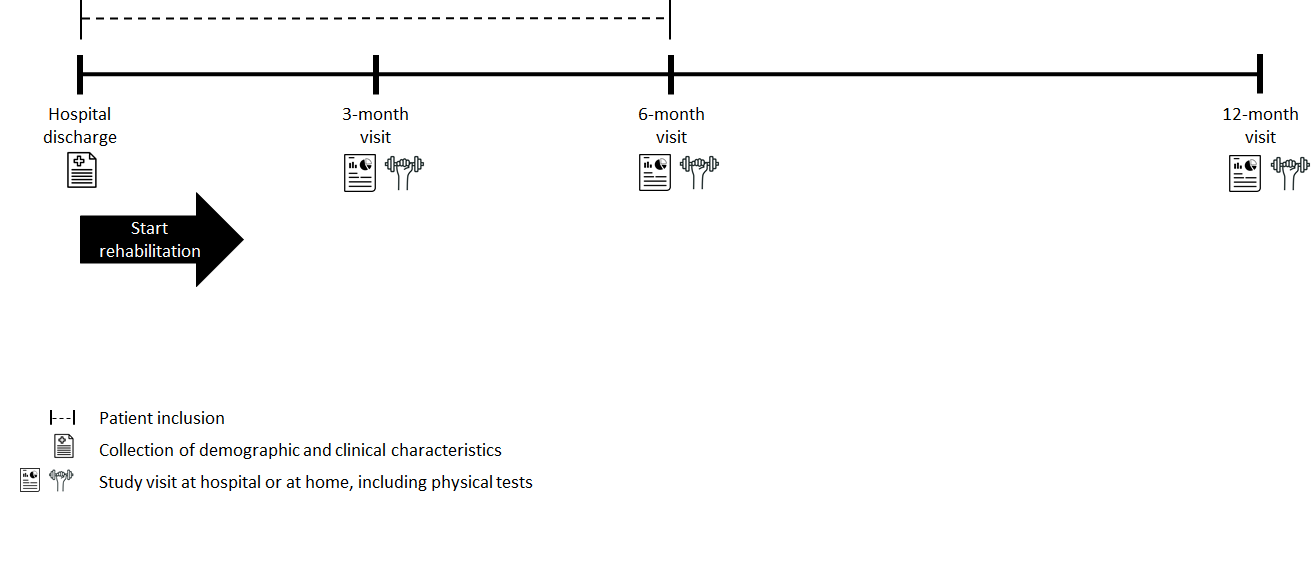
**

**Figure S1. Study timeline.** Study participants were included within 6 months but preferably within 3 months after hospitalization for COVID-19. Study measurements were performed at 3, 6, and 12 months after hospital discharge. Rehabilitation starts after hospital discharge and may contain community-based rehabilitation, in- and/or outpatient medical rehabilitation, or inpatient rehabilitation in a skilled nursing facility.

**Supplementary Figure S2**

**
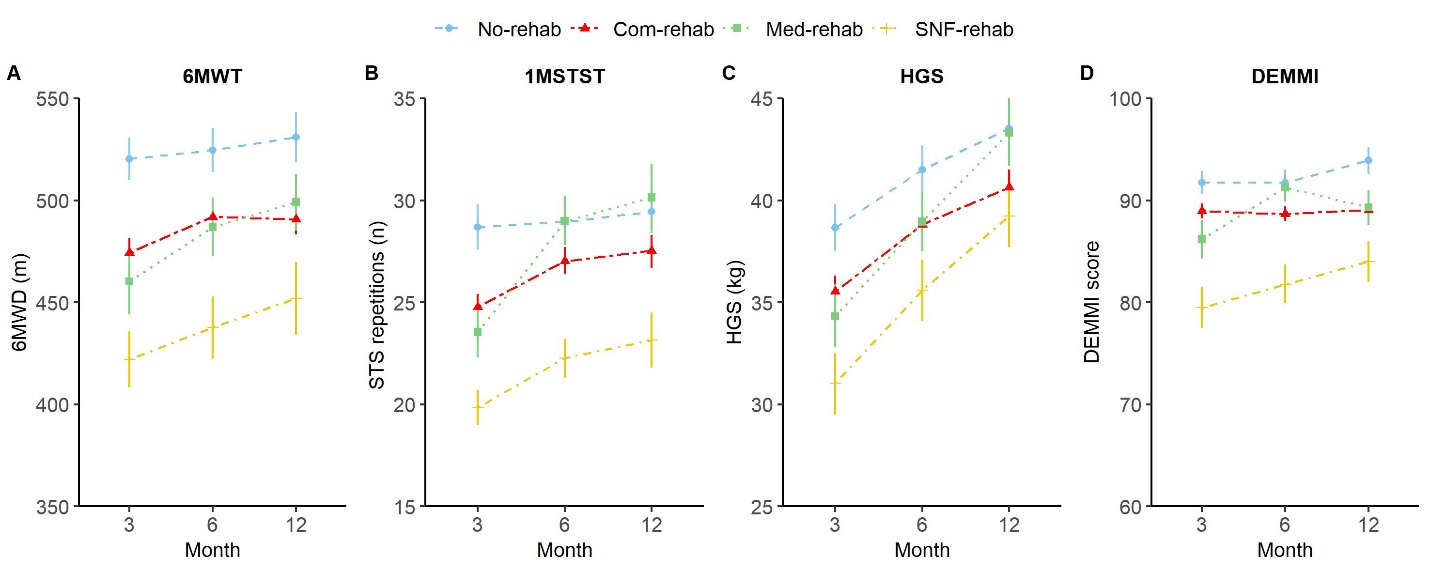
Figure S2. Trajectories of outcomes in 6MWT, 1MSTST, HGS, and DEMMI over time within care pathways assessed at 3, 6, and 12 months after hospital discharge.** Trajectories of physical outcomes over time were assessed using generalized estimating equations analysis. Data are presented as estimated mean with standard error. Care pathways comprise patients with No-rehab: no rehabilitation, Com-rehab: community-based rehabilitation, Med-rehab: in- and outpatient medical rehabilitation, and SNF-rehab: inpatient rehabilitation in a skilled nursing facility after hospitalization for COVID-19. 6MWT=6 min walk test; 6MWD=6 min walk distance; 1MSTST=1 min sit-to-stand test; STS=sit-to-stand; HGS=handgrip strength; DEMMI=de Morton Mobility Index.

**Supplementary Figure S3**

**
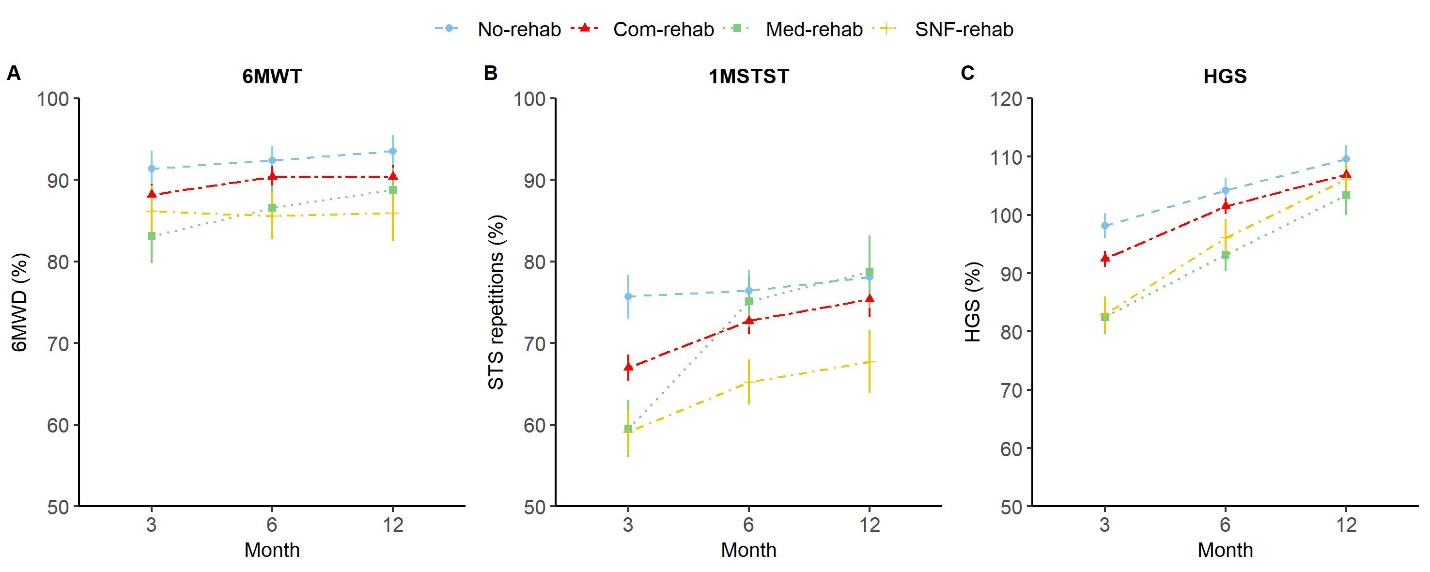
Figure S3. Trajectories of the percentage of normative values reached in 6MWT, 1MSTST, and HGS over time within care pathways assessed at 3, 6, and 12 months after hospital discharge, outcomes of unadjusted generalized estimating equations analysis.** Trajectories of the percentages of normative values reached in physical outcomes over time were assessed using generalized estimating equations. Data are presented as estimated mean with standard error. Care pathways comprise patients with No-rehab: no rehabilitation, Com-rehab: community-based rehabilitation, Med-rehab: in- and outpatient medical rehabilitation, and SNF-rehab: inpatient rehabilitation in a skilled nursing facility after hospitalization for COVID-19. Normative values in 6MWT are calculated using reference equations described by Enright and Sherill,^1^ in 1MSTST using reference values described by Strassman and colleagues,^2^ and in HGS using reference values described by Dodds and colleagues.^3^ 6MWT=6 min walk test; 6MWD=6 min walk distance; 1MSTST=1 min sit-to-stand test; STS=sit-to-stand; HGS=handgrip strength.

**Supplementary Table S1. Demographic and clinical characteristics of study participants included versus not included in analysis. Values are number (percentage) unless stated otherwise**

|  | **nᵃ** | **Study participants included**  **in analysis (n=582)** | **nᵃ** | **Study participants not included in analysis^b^ (n=68)** | **p value** |
| --- | --- | --- | --- | --- | --- |
| **Demographic characteristics** |  |  |  |  |  |
| Median (IQR) age, years | - | 60·0 (53·0-67·0) | - | 60·5 (54·3-70·0) | 0·57 |
| Sex | - |  | - |  | 0·59 |
| Female |  | 181 (31·1) |  | 19 (27·9) |  |
| Male |  | 401 (68·9) |  | 49 (72·1) |  |
| Median (IQR) BMI, kg/m² | 69 | 29·3 (25·7-32·1) | 11 | 28·3 (26·2-32·7) | 0·67 |
| ≥1 comorbidities | - | 473 (81·3) | 11 | 53 (93·0) | 0·028 |
| *Migration background* | 3 |  | 22 |  | 0·54 |
| European |  | 415 (71·7) |  | 31 (67·4) |  |
| Dutch Caribbean |  | 80 (13·8) |  | 8 (17·4) |  |
| Asian |  | 36 (6·2) |  | 3 (6·5) |  |
| Turkish |  | 25 (4·3) |  | 2 (4·3) |  |
| (North) African |  | 23 (4·0) |  | 2 (4·3) |  |
| *Education level* | 7 |  | 26 |  | 0·23 |
| Low |  | 201 (35·0) |  | 20 (47·6) |  |
| Middle |  | 202 (35·1) |  | 13 (31·0) |  |
| High |  | 172 (29·9) |  | 9 (21·4) |  |
| *Smoking status* | 4 |  | 24 |  | 0·63 |
| Never |  | 254 (43·9) |  | 21 (47·7) |  |
| Former |  | 313 (54·2) |  | 22 (50·0) |  |
| Current |  | 11 (1·9) |  | 1 (2·3) |  |
| *Physical activity level*^c^ | 5 |  | 28 |  | 0·26 |
| Inactive |  | 76 (13·2) |  | 8 (20·0) |  |
| Light |  | 305 (52·9) |  | 24 (60·0) |  |
| Regular |  | 159 (27·6) |  | 7 (17·5) |  |
| Hard |  | 37 (6·4) |  | 1 (2·5) |  |
| Employed | 6 | 344 (59·7) | 25 | 20 (46·5) | 0·090 |
| **Clinical characteristics** |  |  |  |  |  |
| ICU admission | - | 237 (40·7) | - | 36 (52·9) | 0·053 |
| Median (IQR) LOS ICU, days | 4 | 16 (9·0-30·0) | - | 24·0 (10·3-46·5) | 0·090 |
| Median (IQR) LOS hospital, days | 1 | 12 (6·0-27·0) | - | 16·0 (7·0-40·0) | 0·012 |

P value was obtained using a χ2 test or Kruskal-Wallis test, as appropriate. IQR: interquartile range. ^a^ In case of missing data the number of patients with missing data are presented. ^b^ Study participants not included in analysis comprise patients who did not attended physical tests during one of the follow-up visits at 3, 6, or 12 months after hospital discharge, including patients who only performed study questionnaires, withdrawn before the start of study measurements, deceased, and those who were lost to follow-up. ^c^ Leisure time physical activity level was measured with the Saltin-Grimby Physical Activity Level Scale questionnaire.^4^

**Supplementary Table S2. Physical function at 3, 6, 12 months after hospital discharge, source data. Values are median (interquartile range) unless stated otherwise**

|  | 3 months | 6 months | 12 months |
| --- | --- | --- | --- |
| Cardiorespiratory fitness |  |  |  |
| *6MWT, n* | 398 | 453 | 239 |
| 6MWD, m | 494·0 (422·0-556·3) | 507·0 (435·0-570·0) | 520·0 (445·0-572·0) |
| 6MWD, %pred^a^ | 89·4 (78·2-101·4) | 91·3 (79·8-103·8) | 92·9 (80·7-105·6) |
| Number (%) breaks or premature stop | 22 (5·6) | 19 (4·2) | 14 (5·9) |
| Number (%) <LLN^a^ | 81 (21·4) | 73 (16·5) | 40 (16·8) |
| Number (%) oxygen desaturation ≥ 4% | 99 (25·3) | 96 (21·9) | 52 (22·1) |
| *1MSTST, n* | 414 | 493 | 273 |
| STS repetitions, n | 24·0 (19·0-30·0) | 26·0 (20·0-33·0) | 26·0 (20·0-34·0) |
| STS repetitions, %pred^b^ | 63·6 (51·5-82·1) | 68·8 (55·6-87·7) | 74·1 (56·7-93·3) |
|  |  |  |  |
| Muscle strength |  |  |  |
| *HGS, n* | 442 | 512 | 280 |
| Maximum HGS, kg | 37·0 (26·0-46·0) | 39·0 (28·0-49·0) | 38·5 (30·0-50·0) |
| Maximum HGS, %pred^c^ | 94·6 (76·7-109·1) | 101·8 (84·3-116·9) | 107·6 (90·7-121·4) |
| Number (%) weak HGS | 51 (11·5) | 32 (6·3) | 16 (5·7) |
|  |  |  |  |
| Mobility |  |  |  |
| *DEMMI, n* | 419 | 493 | 270 |
| Total score | 85·0 (85·0-100·0) | 85·0 (85·0-100·0) | 100·0 (85·0-100·0) |

The number of data points at each time point is presented per physical test. 6MWT=6 min walk test; 6MWD=6 min walk distance; %pred=percentage of normative values; <LLN=below the lower limit of normal; 1MSTST=1 min sit-to-stand test; STS: sit-to-stand; HGS=handgrip strength; DEMMI=de Morton Mobility Index. ^a^ Calculated using reference equations described by Enright and Sherill.^1^ ^b^ Reference values described by Strassman and colleagues.^2^ ^c^ Reference values described by Dodds and colleagues.^3^

**Supplementary Table S3.** **Physical function across care pathways at 3, 6, and 12 months after hospital discharge, source data.**

|  | **No-rehab**  **(n = 114)** | | | **Com-rehab**  **(n = 315)** | | | **Med-rehab**  **(n = 80)** | | | **SNF-rehab**  **(n = 73)** | | |
| --- | --- | --- | --- | --- | --- | --- | --- | --- | --- | --- | --- | --- |
|  | **3**  **months** | **6**  **months** | **12**  **months** | **3**  **months** | **6**  **months** | **12**  **months** | **3**  **months** | **6**  **months** | **12**  **months** | **3**  **months** | **6**  **months** | **12**  **months** |
| **Cardiorespiratory fitness** | | | | | | | | | | | | |
| *6MWT, n* | 77 | 85 | 54 | 236 | 246 | 118 | 39 | 62 | 37 | 46 | 60 | 30 |
| 6MWD, m | 525·0  (459·0-601·5) | 530·0  (462·5-606·5) | 559·0  (503·8-617·5) | 489·5 (423·5-553·8) | 510·0 (445·0-575·0) | 510·0 (443·8-561·3) | 510·0 (350·0-555·0) | 512·5 (418·0-556·0) | 510·0 (430·0-547·0) | 427·0 (375·0-194·3) | 445·0 (382·5-535·0) | 446·0 (357·5-522·5) |
| 6MWD, %pred^a^ | 90·2  (82·2-103·2) | 92·8  (80·8-104·9) | 92·8  (82·1-108·6) | 89·3  (78·5-102·4) | 93·0  (80·3-104·6) | 94·7  (79·6-106·1) | 87·5  (67·6-100·0) | 86·2  (77·2-102·6) | 94·6  (82·0-103·7) | 87·0  (73·1-97·8) | 88·3  (72·6-100·6) | 91·0  (79·5-100·0) |
| Number (%) breaks or premature stop | 2  (2·6) | 3  (3·5) | 3  (5·6) | 15  (6·4) | 7  (2·8) | 3  (5·6) | 5  (4·2) | 3  (4·8) | 3  (9·1) | 3  (8·1) | 6  (10·2) | 3  (10·0) |
| <LLN^a^ | 9  (12·3) | 11  (13·3) | 10  (18·5) | 51  (22·5) | 36  (14·9) | 21  (17·9) | 11  (32·4) | 13  (22·0) | 5  (13·5) | 10  (22·2) | 13  (22·0) | 4  (13·3) |
| Number (%) oxygen desaturation  ≥ 4% | 14  (18·9) | 16  (19·5) | 10  (18·9) | 51  (21·8) | 36  (15·1) | 24  (20·7) | 18  (47·4) | 24  (40·7) | 12  (33·3) | 16  (35·6) | 20  (34·5) | 6  (20·0) |
| *1MSTST, n* | 85 | 95 | 57 | 240 | 268 | 142 | 41 | 65 | 41 | 48 | 65 | 33 |
| STS repetitions, n | 28·0  (22·5-35·5) | 27·0  (21·0-27·0) | 29·0 (23·5-36·0) | 23·0 (19·0-30·0) | 25·0 (20·0-33·0) | 25·0 (20·0-34·0) | 24·0 (20·0-32·0) | 28·0 (21·5-34·0) | 29·0 (21·5-41·5) | 19·0 (15·0-24·0) | 21·0 (17·5-28·0) | 24·0 (18·0-28·5) |
| STS repetitions, %pred^b^ | 75·6  (58·8-93·2) | 71·4  (57·1-90·2) | 83·8  (59·7-96·1) | 63·4  (51·4-79·7) | 68·6  (55·9-87·3) | 69·7  (54·1-93·4 | 62·9  (48·8-78·0) | 68·6  (58·3-93·2) | 76·2  (60·0-106·9) | 58·1  (41·8-75·0) | 65·6  (51·4-82·4) | 68·9  (54·0-84·9) |

|  | **No-rehab**  **(n = 114)** | | | **Com-rehab**  **(n = 315)** | | | **Med-rehab**  **(n = 80)** | | | **SNF-rehab**  **(n = 73)** | | |
| --- | --- | --- | --- | --- | --- | --- | --- | --- | --- | --- | --- | --- |
|  | **3**  **months** | **6**  **months** | **12**  **months** | **3**  **months** | **6**  **months** | **12**  **months** | **3**  **months** | **6**  **months** | **12**  **months** | **3**  **months** | **6**  **months** | **12**  **months** |
| **Muscle strength** | | | | | | | | | | | | |
| *HGS, n* | 87 | 97 | 59 | 253 | 276 | 143 | 46 | 68 | 42 | 56 | 71 | 36 |
| Maximum HGS, kg | 40·0  (30·0-50·0) | 41·0  (30·5-52·0) | 45·0  (32·0-57·0) | 36·0  (25·0-46·0) | 39·0  (28·0-49·0) | 38·0  (29·0-48·0) | 37·0  (26·5-46·0) | 38·5  (31·0-46·0) | 37·5  (32·8-52·0) | 28·0  (23·3-38·8) | 36·0  (25·0-45·0) | 38·0  (26·3-49·3) |
| Maximum HGS, %pred^c^ | 100·8 (87·4-110·7) | 105·7 (88·7-118·7) | 109·4 (100·9-125·6) | 94·5  (77·8-109·9) | 103·1 (87·0-117·6) | 107·6 (89·5-122·0) | 86·6  (59·0-99·6) | 90·9  (75·9-111·7) | 100·2 (80·1-117·3) | 83·2  (66·4-105·7) | 93·6  (76·7-115·2) | 107·1 (94·0-119·1) |
| Number (%) weak HGS | 4  (4·6) | 4  (4·1) | 2  (3·4) | 23  (9·1) | 11  (4·0) | 8  (5·6) | 11  (23·9) | 6  (8·8) | 3  (7·1) | 13  (22·8) | 11  (15·3) | 3  (8·3) |
| **Mobility** | | | | | | | | | | | | |
| *DEMMI, n* | 81 | 93 | 55 | 239 | 266 | 140 | 46 | 63 | 40 | 53 | 71 | 35 |
| Total score | 100·0 (85·0-100·0) | 100·0 (85·0-100·0) | 100·0 (100·0-100·0) | 85·0 (85·0-100·0) | 85·0 (85·0-100·0) | 85·0 (85·0-100·0) | 85·0 (85·0-100·0) | 100·0 (85·0-100·0) | 92·5 (85·0-100·0) | 85·0 (74·0-85·0) | 85·0 (74·0-100·0) | 85·0 (74·0-100·0) |

Data are presented as median (interquartile range) unless stated otherwise. The number of data points at each time point is presented across care pathways per physical test. Care pathways comprise patients with No-rehab: no rehabilitation, Com-rehab: community-based rehabilitation, Med-rehab: in- and outpatient medical rehabilitation, and SNF-rehab: inpatient rehabilitation in a skilled nursing facility after hospitalization for COVID-19. 6MWT=6 min walk test; 6MWD=6 min walk distance; %pred=percentage of normative values; 1MSTST=1 min sit-to-stand test; STS: sit-to-stand; HGS=handgrip strength; DEMMI=de Morton Mobility Index. ^a^ Calculated using reference equations described by Enright and Sherill.^1^ ^b^ Reference values described by Strassman and colleagues.^2^ ^c^ Reference values described by Dodds and colleagues.^3^

**Supplementary Table S4. 6MWT outcomes across care pathways at 3, 6, and 12 months after hospital discharge.**

|  | **3 months** | **6 months** | **12 months** | **Mean difference 3 to 6 months (95% confidence interval); p value** | **Mean difference 6 to 12 months (95% confidence interval); p value** | **Mean difference 3 to 12 months (95% confidence interval); p value** |
| --- | --- | --- | --- | --- | --- | --- |
| **6MWD, m** |  |  |  |  |  |  |
| No-rehab | 488·1 (15·6) | 496·3 (14·5) | 504·6 (15·7) | 8·2 (-12·5 to 28·9); 0·4 | 8·4 (-7·9 to 24·6); 0·3 | 16·6 (-4·3 to 37·4); 0·1 |
| Com-rehab | 464·5 (13·2) | 478·6 (13·4) | 479·8 (13·8) | 14·1 (3·3 to 24·9); 0·01 | 1·2 (-10·6 to 13·1); 0·8 | 15·3 (0·9 to 29·7); 0·04 |
| Med-rehab | 463·6 (23·8) | 491·4 (22·8) | 503·8 (22·8) | 27·9 (0·4 to 55·3); 0·047 | 12·4 (-6·2 to 31·0); 0·2 | 40·3 (11·0 to 69·5); 0·007 |
| SNF-rehab | 442·8 (20·8) | 443·7 (19·9) | 452·2 (23·0) | 0·9 (-29·5 to 31·3); 1·0 | 8·4 (-18·7 to 35·6); 0·5 | 9·3 (-30·8 to 49·5); 0·6 |
| **6MWD, %pred** |  |  |  |  |  |  |
| No-rehab | 92·2 (2·9) | 93·5 (2·7) | 94·8 (3·0) | 1·2 (-2·5 to 5·0); 0·5 | 1·3 (-1·7 to 4·3); 0·4 | 2·5 (-1·0 to 6·0); 0·2 |
| Com-rehab | 89·6 (2·4) | 91·9 (2·4) | 91·9 (2·4) | 2·4 (0·2 to 4·6); 0·03 | -0·0007 (-2·4 to 2·4); 1·0 | 2·4 (-0·5 to 5·3); 0·01 |
| Med-rehab | 90·0 (4·5) | 93·4 (4·3) | 95·7 (4·1) | 3·4 (-2·3 to 9·1); 0·2 | 2·3 (-1·5 to 6·1); 0·2 | 5·7 (-0·8 to 12·2); 0·08 |
| SNF-rehab | 89·2 (3·9) | 88·6 (3·7) | 89·0 (4·6) | -0·6 (-6·5 to 5·3); 0·8 | 0·4 (-5·4 to 6·2); 0·9 | -0·2 (-8·6 to 8·2); 1·0 |

Data are presented as estimated mean (standard error) unless stated otherwise, obtained from generalized estimating equations analysis. Number of observations within No-rehab: 213, Com-rehab: 597, Med-rehab: 133, and SNF-rehab: 134. Care pathways comprise patients with No-rehab: no rehabilitation, Com-rehab: community-based rehabilitation, Med-rehab: in- and outpatient medical rehabilitation, and SNF-rehab: inpatient rehabilitation in a skilled nursing facility after hospitalization for COVID-19. Normative values were calculated using reference equations described by Enright and Sherill.^1^ Generalized estimating equations analysis was performed to assess the trajectories of 6MWD (in meters) over time within care pathways, adjusted for demographic and clinical characteristics during hospital admission for COVID-19 including age (p=0·2), sex (male versus female; p<0·001), having one or more comorbidities (no versus yes; p=0·01), obesity (no versus yes [body mass index ≥30 kg/m^2^]; p=0·5), employed (no versus yes; p=0·3), delirium (no or unknown versus yes; p=0·3), thrombotic event (no or unknown versus yes; p=1·0), admission to intensive care unit (no versus yes; p=0·04), and the length of hospital stay (days, p=0·003). Likewise, a second generalized estimating equations analysis was performed to assess the trajectories of the percentages of normative values reached in 6MWD within care pathways, adjusted for having one or more comorbidities (no versus yes; p=0·04), employed (no versus yes; p=0·4), delirium (no or unknown versus yes; p=0·2), thrombotic event (no or unknown versus yes; p=0·9), admission to intensive care unit (no versus yes; p=0·002), and the length of hospital stay (days, p=0·003); age, sex, and obesity were excluded (normative values are already adjusted for these characteristics). 6MWT=6 min walk test; 6MWD=6 min walk distance; %pred=percentage of normative value.

**Supplementary Table S5. 1MSTST outcomes across care pathways at 3, 6, and 12 months after hospital discharge.**

|  | **3 months** | **6 months** | **12 months** | **Mean difference 3 to 6 months (95% confidence interval); p value** | **Mean difference 6 to 12 months (95% confidence interval); p value** | **Mean difference 3 to 12 months (95% confidence interval); p value** |
| --- | --- | --- | --- | --- | --- | --- |
| **STS repetitions, n** |  |  |  |  |  |  |
| No-rehab | 27·9 (1·2) | 28·3 (1·1) | 28·8 (1·1) | 0·4 (-1·2 to 1·9); 0·7 | 0·5 (-0·9 to 1·8); 0·5 | 0·9 (-1·1 to 2·8); 0·4 |
| Com-rehab | 25·4 (1·0) | 27·7 (1·0) | 28·2 (1·1) | 2·3 (1·4 to 3·2); <0·001 | 0·5 (-0·8 to 1·7); 0·5 | 2·8 (1·4 to 4·2); <0·001 |
| Med-rehab | 23·0 (1·8) | 28·6 (1·6) | 29·8 (2·0) | 5·6 (3·9 to 7·2); <0·001 | 1·2 (-1·6 to 4·0); 0·4 | 6·8 (3·4 to 10·2); <0·001 |
| SNF-rehab | 21·5 (1·3) | 23·9 (1·3) | 24·7 (1·5) | 2·3 (0·9 to 3·8); 0·002 | 0·9 (-1·4 to 3·1); 0·5 | 3·2 (1·1 to 5·2); 0·002 |
| **STS repetitions, %pred** |  |  |  |  |  |  |
| No-rehab | 78·5 (3·1) | 79·6 (2·9) | 81·2 (3·1) | 1·1 (-3·2 to 5·4); 0·6 | 1·6 (-1·9 to 5·1); 0·4 | 2·7 (-2·4 to 7·8); 0·3 |
| Com-rehab | 71·5 (2·7) | 77·8 (2·8) | 80·1 (3·3) | 6·3 (3·8 to 8·7); <0·001 | 2·3 (-1·3 to 6·0); 0·2 | 8·6 (4·7 to 12·5); <0·001 |
| Med-rehab | 66·6 (4·6) | 80·7 (4·4) | 84·2 (5·4) | 14·1 (10·1 to 18·1); <0·001 | 3·5 (-3·8 to 10·8); 0·3 | 17·6 (9·1 to 26·1); <0·001 |
| SNF-rehab | 63·0 (3·5) | 69·9 (3·6) | 72·7 (4·3) | 6·9 (2·6 to 11·0); 0·001 | 2·8 (-4·2 to 9·7); 0·4 | 9·6 (3·2 to 16·1); 0·003 |

Data are presented as estimated mean (standard error) unless stated otherwise, obtained from generalized estimating equations analysis. Number of observations within No-rehab: 235, Com-rehab: 646, Med-rehab: 143, and SNF-rehab: 144. Care pathways comprise patients with No-rehab: no rehabilitation, Com-rehab: community-based rehabilitation, Med-rehab: in- and outpatient medical rehabilitation, and SNF-rehab: inpatient rehabilitation in a skilled nursing facility after hospitalization for COVID-19. Normative values were calculated using reference values described by Strassman and colleagues.^2^ Generalized estimating equations analysis was performed to assess the trajectories of 1MSTST outcome (number of STS repetitions) over time within care pathways, adjusted for demographic and clinical characteristics during hospital admission for COVID-19 including age (p<0·001), sex (male versus female; p<0·001), having one or more comorbidities (no versus yes; p<0·001), obesity (no versus yes [body mass index ≥30 kg/m^2^]; p=0·5), employed (no versus yes; p=0·009), delirium (no or unknown versus yes; p=0·2), thrombotic event (no or unknown versus yes; p=0·4), admission to intensive care unit (no versus yes; p=0·001), and the length of hospital stay (days, p=0·05). Likewise, a second generalized estimating equations analysis was performed to assess the trajectories of the percentages of normative values reached in STS repetitions within care pathways, adjusted for obesity (no versus yes [body mass index ≥30 kg/m^2^]; p=0·07), having one or more comorbidities (no versus yes; p<0·001), employed (no versus yes; p=0·6), delirium (no or unknown versus yes; p=0·2), thrombotic event (no or unknown versus yes; p=0·2), admission to intensive care unit (no versus yes; p=0·003), and the length of hospital stay (days, p=0·1); age and sex were excluded (normative values are already adjusted for these characteristics). 1MSTST=1 min sit-to-stand test; STS: sit-to-stand; %pred=percentage of normative value.

**Supplementary Table S6. HGS outcomes across care pathways at 3, 6, and 12 months after hospital discharge.**

|  | **3 months** | **6 months** | **12 months** | **Mean difference 3 to 6 months (95% confidence interval); p value** | **Mean difference 6 to 12 months (95% confidence interval); p value** | **Mean difference 3 to 12 months (95% confidence interval); p value** |
| --- | --- | --- | --- | --- | --- | --- |
| **Maximum HGS, kg** |  |  |  |  |  |  |
| No-rehab | 32·1 (1·0) | 34·8 (1·0) | 36·8 (1·1) | 2·7 (1·5 to 3·9); <0·001 | 2·0 (0·7 to 3·2); 0·002 | 4·6 (3·0 to 6·3); <0·001 |
| Com-rehab | 31·6 (0·8) | 35·0 (0·8) | 36·8 (0·8) | 3·4 (2·7 to 4·2); <0·001 | 1·8 (0·9 to 2·7); <0·001 | 5·2 (4·2 to 6·2); <0·001 |
| Med-rehab | 30·2 (1·6) | 34·5 (1·5) | 39·2 (1·7) | 4·3 (2·3 to 6·4); <0·001 | 4·7 (3·1 to 6·2); <0·001 | 9·0 (6·7 to 11·4); <0·001 |
| SNF-rehab | 30·3 (1·3) | 34·9 (1·3) | 38·5 (1·3) | 4·5 (3·1 to 6·0); <0·001 | 3·6 (1·8 to 5·5); <0·001 | 8·2 (5·9 to 10·5); <0·001 |
| **Maximum HGS, %pred** |  |  |  |  |  |  |
| No-rehab | 96·1 (2·5) | 102·0 (2·4) | 107·3 (2·7) | 6·0 (2·9 to 9·0); <0·001 | 5·3 (1·9 to 8·7); 0·002 | 11·3 (7·1 to 15·4); <0·001 |
| Com-rehab | 91·9 (2·0) | 100·9 (1·9) | 106·4 (2·1) | 9·1 (7·1 to 11·0); <0·001 | 5·5 (2·9 to 8·0); <0·001 | 14·5 (11·9 to 17·2); <0·001 |
| Med-rehab | 88·6 (4·1) | 99·0 (4·0) | 109·9 (4·4) | 10·4 (5·6 to 15·1); <0·001 | 10·9 (7·1 to 14·8); <0·001 | 21·3 (15·4 to 27·2); <0·001 |
| SNF-rehab | 88·2 (3·6) | 101·3 (3·8) | 111·5 (3·6) | 13·2 (9·5 to 16·9); <0·001 | 10·1 (5·0 to 15·3); <0·001 | 23·3 (17·1 to 29·6); <0·001 |

Data are presented as estimated mean (standard error) unless stated otherwise, obtained from generalized estimating equations analysis. Number of observations within No-rehab: 241, Com-rehab: 668, Med-rehab: 151, and SNF-rehab: 161. Care pathways comprise patients with No-rehab: no rehabilitation, Com-rehab: community-based rehabilitation, Med-rehab: in- and outpatient medical rehabilitation, and SNF-rehab: inpatient rehabilitation in a skilled nursing facility after hospitalization for COVID-19. Normative values were calculated using reference values described by Dodds and colleagues.^3^ Generalized estimating equations analysis was performed to assess the trajectories of maximum HGS (in kg) over time within care pathways, adjusted for demographic and clinical characteristics during hospital admission for COVID-19 including age (p<0·001), sex (male versus female; p<0·001), having one or more comorbidities (no versus yes; p=0·1), obesity (no versus yes [body mass index ≥30 kg/m^2^]; p=0·7), employed (no versus yes; p<0·001), delirium (no or unknown versus yes; p=0·8), thrombotic event (no or unknown versus yes; p=0·4), admission to intensive care unit (no versus yes; p=0·5), and the length of hospital stay (days, p=0·002). Likewise, a second generalized estimating equations analysis was performed to assess the trajectories of the percentages of normative values reached in HGS within care pathways, adjusted for obesity (no versus yes [body mass index ≥30 kg/m^2^]; p=0·5), having one or more comorbidities (no versus yes; p=0·09), employed (no versus yes; p=0·03), delirium (no or unknown versus yes; p=0·5), thrombotic event (no or unknown versus yes; p=0·7), admission to intensive care unit (no versus yes; p=0·4), and the length of hospital stay (days, p=0·02); age and sex were excluded (normative values are already adjusted for these characteristics). HGS: handgrip strength; %pred=percentage of normative value.

**Supplementary Table S7. DEMMI score across care pathways at 3, 6, and 12 months after hospital discharge. Results from generalized estimating equations analysis, values are estimated mean (standard error) unless stated otherwise**

|  | **3 months** | **6 months** | **12 months** | **Mean difference 3 to 6 months (95% confidence interval); p value** | **Mean difference 6 to 12 months (95% confidence interval); p value** | **Mean difference 3 to 12 months (95% confidence interval); p value** |
| --- | --- | --- | --- | --- | --- | --- |
| **DEMMI, total score** |  |  |  |  |  |  |
| No-rehab | 91·2 (1·3) | 91·3 (1·4) | 93·0 (1·4) | 0·1 (-2·0 to 2·3); 0·9 | 1·7 (-0·4 to 3·9); 0·1 | 1·8 (-0·6 to 4·2); 0·1 |
| Com-rehab | 89·4 (1·0) | 89·0 (1·0) | 89·4 (1·2) | -0·4 (-1·7 to 0·9); 0·6 | 0·3 (-1·4 to 2·0); 0·7 | -0·04 (-1·9 to 1·8); 1·0 |
| Med-rehab | 83·7 (2·3) | 89·8 (1·9) | 87·2 (2·3) | 6·1 (3·0 to 9·2); <0·001 | -2·6 (-6·4 to 1·1); 0·2 | 3·5 (-1·1 to 8·0); 0·1 |
| SNF-rehab | 80·7 (2·1) | 83·0 (1·9) | 85·5 (2·0) | 2·3 (-0·9 to 5·6); 0·2 | 2·5 (-1·0 to 6·0); 0·2 | 4·8 (1·1 to 8·6); 0·01 |

Number of observations within No-rehab: 227, Com-rehab: 641, Med-rehab: 144, and SNF-rehab:157. Care pathways comprise patients with No-rehab: no rehabilitation, Com-rehab: community-based rehabilitation, Med-rehab: in- and outpatient medical rehabilitation, and SNF-rehab: inpatient rehabilitation in a skilled nursing facility after hospitalization for COVID-19. Generalized estimating equations analysis was performed to assess the trajectories of DEMMI score over time within care pathways, adjusted for demographic and clinical characteristics during hospital admission for COVID-19 including age (p<0·001), sex (male versus female; p=0·02), having one or more comorbidities (no versus yes; p=0·2), obesity (no versus yes [body mass index ≥30 kg/m^2^]; p<0·001), employed (no versus yes; p=0·01), delirium (no or unknown versus yes; p=0·9), thrombotic event (no or unknown versus yes; p=0·4), admission to intensive care unit (no versus yes; p<0·001), and the length of hospital stay (days, p=0·05). DEMMI: de Morton Mobility Index.

**References**

1. Enright PL, Sherrill DL. Reference equations for the six-minute walk in healthy adults. American journal of respiratory and critical care medicine. 1998;158(5):1384-7.

2. Strassmann A, Steurer-Stey C, Dalla Lana K, et al. Population-based reference values for the 1-min sit-to-stand test. International journal of public health. 2013;58(6):949-53.

3. Dodds RM, Syddall HE, Cooper R, et al. Grip strength across the life course: normative data from twelve British studies. PloS one. 2014;9(12):e113637.

4. Grimby G, Börjesson M, Jonsdottir IH, Schnohr P, Thelle DS, Saltin B. The “Saltin–Grimby physical activity level scale” and its application to health research. Scandinavian journal of medicine & science in sports 2015; 25: 119-25.
